# Light structuring via nonlinear total angular momentum addition with flat optics

**DOI:** 10.1038/s41377-025-02004-8

**Published:** 2025-11-12

**Authors:** Evgenii Menshikov, Paolo Franceschini, Kristina Frizyuk, Ivan Fernandez-Corbaton, Andrea Tognazzi, Alfonso Carmelo Cino, Denis Garoli, Mihail Petrov, Domenico de Ceglia, Costantino De Angelis

**Affiliations:** 1 Department of Information Engineering, University of Brescia, Via Branze, 38, Brescia, 25123 Italy; 2 National Institute of Optics-National Research Council, Via Branze, 45, Brescia, 25123 Italy; 3 Karlsruhe Institute of Technology, Kaiserstrasse, 12, Karlsruhe, 76131 Germany; 4 Department of Engineering, University of Palermo, Viale delle Scienze, 9, Palermo, 90128 Italy; 5 Dipartimento di Scienze e Metodi dell’Ingegneria, Università Degli Studi di Modena e Reggio Emilia, Via Amendola, 2, Reggio Emilia, 43122 Italy; 6 New Uzbekistan University, Mirzo Ulugbek Movarounnahr 1, Tashkent, Uzbekistan

**Keywords:** Nonlinear optics, Optical metrology

## Abstract

Shaping the structure of light with flat optical devices has driven significant advancements in our fundamental understanding of light and light-matter interactions, and enabled a broad range of applications, from image processing and microscopy to optical communication, quantum information processing, and the manipulation of microparticles. Yet, pushing the boundaries of structured light beyond the linear optical regime remains an open challenge. Nonlinear optical interactions, such as wave mixing in nonlinear flat optics, offer a powerful platform to unlock new degrees of freedom and functionalities for generating and detecting structured light. In this study, we experimentally demonstrate the non-trivial structuring of third-harmonic light enabled by the addition of total angular momentum projection in a nonlinear, isotropic flat optics element—a single thin film of amorphous silicon. We identify the total angular momentum projection and helicity as the most critical properties for analyzing the experimental results. The theoretical approach we propose, supported by numerical simulations, offers quantitative predictions for light structuring through nonlinear wave mixing under various pumping conditions, including vectorial and non-paraxial pump light. Notably, we reveal that the shape of third-harmonic light is highly sensitive to the polarization state of the pump. Our findings demonstrate that harnessing the addition of total angular momentum projection in nonlinear wave mixing can be a powerful strategy for generating and detecting precisely controlled structured light.

## Introduction

Light beams with carefully engineered spatial structures can carry various transverse modes, offering an extraordinary resource when harnessed effectively^1^. This has ignited a surge of interest in the field of structured light, leading to numerous applications across diverse areas such as image processing^2,3^, superresolution microscopy^4–6^, metrology^7,8^, communication^9^, quantum information processing^10,11^, and light–matter interactions^12,13^. Furthermore, structured light in nonlinear optical devices shows great promise for advancing analog deep neural networks, where the vast number of modes can be leveraged to enhance parallel processing and data encoding capabilities^14,15^. Recently, a relevant paradigm shift has occurred in the light shaping field, driven by advancements in flat optics and photonic nanostructures^16–21^.

In contrast to linear optics, which typically influences only a single degree of freedom, nonlinear processes such as frequency mixing possess the fascinating ability to interconnect multiple degrees of freedom via the characteristics of the medium (the shape and symmetries of the nonlinear susceptibility tensor, in addition to the shape and symmetry of the structure)^22–24^ (see also the review in ref. ^25^, along with references therein). In flat optics, metasurfaces are widely used to generate various types of structured light. On the one hand, they provide more degrees of freedom, allowing for complex light tailoring; on the other hand, they are more demanding in terms of fabrication^26–29^.

To fully exploit the potential in the above-mentioned applications, a powerful and yet simple approach to describe the input-output relationships in real devices is needed. Very often, to simplify this task, which involves vector fields, one tries to construct some scalar description of the problem. In some cases, however, considering the vector field as a whole can actually simplify matters when one exploits the symmetry properties of both the light field and the material systems. For example, considering the behavior of the fields under rotations about a particular axis is relevant for samples with cylindrical symmetry. Such behavior is determined by the projection of the total angular momentum (TAM) of the field on the optical axis^30^. Another property of the fields that helps in the analysis of experiments^31–33^ is the handedness of light, or helicity^34–37^. Both total angular momentum and helicity are fundamental quantities of the electromagnetic field, which are connected to the symmetries of Maxwell’s equations (see Suppl. Inf. A for definitions). Their analysis allows one to readily predict some aspects of the light–matter interaction using the conservation laws related to the particular symmetries of the system. Both quantities are generally valid for electromagnetic fields, independently of whether the fields are collimated, focused, far fields, or evanescent. Moreover, symmetry analysis enables studying and predicting spontaneous parametric down-conversion (SPDC) from thin films, because this process is governed by similar selection rules and the same mechanism^38,39^.

There are well-known examples of fields that transform under rotations in particularly simple ways: the vector spherical harmonics, which are spherical waves describing the electric and magnetic fields of multipoles^30,40,41^ (well-defined TAM and TAM projection), or the Bessel beams^42^, which are cylindrical waves (well-defined TAM projection). With respect to helicity, one may obtain purely circularly polarized (CP) spherical or cylindrical waves by linear combinations of electric and magnetic multipolar fields^36^, or TE/TM Bessel beams^43^.

**Fig. 1:**
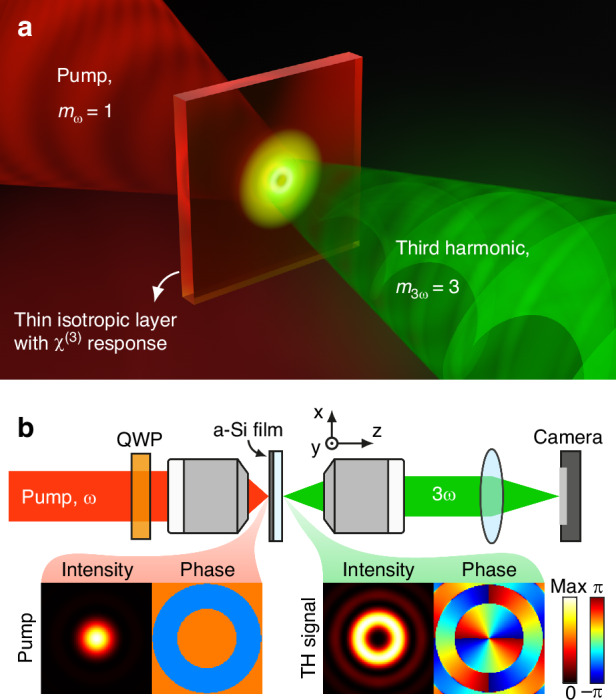
Nonlinear total angular momentum z-projection addition. **a** Illustration of the nonlinear total angular momentum (TAM) $z$-projection ($m$) addition process. When a circularly polarized (CP) pump beam (${m}_{\omega }=1$) is incident on a thin isotropic layer with ${\chi }^{\left(3\right)}$ nonlinearity, the generated signal at third-harmonic (TH) frequency has tripled ${\rm{TAM}}$ (${m}_{3\omega }=3$). **b** Schematic of the optical setup for observation of the nonlinear TAM projection addition in a thin film of a-Si. Bottom panel shows numerical simulations of the intensity, ${\left|{\bf{E}}\cdot {{\bf{e}}}_{{{R}}}^{* }\right|}^{2}$, and phase, $\arg \left({\bf{E}}\cdot {{\bf{e}}}_{{{R}}}^{* }\right)$, distributions of the dominant polarization components in the right CP pump and in the generated TH signal on the thin film surface

In this work, we experimentally demonstrate the tripling of the total angular momentum projection through third-order nonlinearity in an amorphous silicon flat film, as schematically shown in Fig. 1. We show that our experimental findings can be explained within the framework of Bessel beams of well-defined helicity. Based on a description of rotational properties of the system by the total angular momentum, the framework offers a simple approach for theoretical analysis of nonlinear generation of non-paraxial fields.

The rest of the article is organized as follows. In the section “Experimental measurements”, we present the results of experimental measurements of third-harmonic signal distributions obtained under tightly focused excitation with various polarization parameters. Section “Theoretical setting” presents the theoretical framework that describes our findings. First, we introduce a straightforward and comprehensive explanation leveraging the rotational symmetry of our system. After that, in the section “Numerical simulation,” we provide a numerical model that produces results closely aligned with the experimental observations. Finally, the section “Helicity change” in the TH simulation discusses the helicity change in nonlinear generation processes.

## Results

### Experimental measurements

We study third-harmonic (TH) generation in amorphous silicon (a-Si) thin films deposited on a fused silica (SiO_2_) substrate (see “Methods” for details). The choice of the substrate material is motivated by its transparency at both the pump and TH wavelengths, and relatively low nonlinear properties, allowing one to neglect the contribution of the substrate to the detected TH signal (${\chi }^{\left(3\right)}$ of a-Si is 4 orders of magnitude larger than that of SiO_2_^44,45^). A simplified schematic of our optical setup is shown in Fig. 1b. Here, a pump beam with a wavelength of 1500 nm is focused on the surface of the a-Si film using an objective lens with high numerical aperture (NA = 0.85). The polarization of the pump beam can be adjusted by changing the orientation of the fast axis of a quarter-wave plate (QWP) mounted on a motorized rotation stage, allowing for excitation with polarizations ranging from linear to nearly circular. The generated TH signal is collected by an objective lens and imaged onto the camera sensor using a tube lens.

**Fig. 2:**
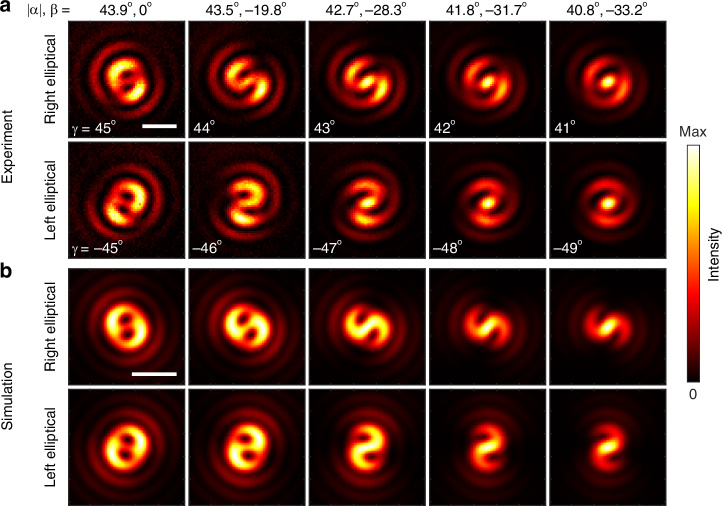
Structuring of third-harmonic light via addition of total angular momentum projection. **a** Optical images of patterns at TH excited with an elliptically polarized pump laser. Here, *α* and *β* denote the ellipticity angle and the inclination angle of the polarization ellipse, respectively, for the pump beam entering the objective lens; *γ* is the angle between the fast axis of the QWP and the polarization direction of the input laser beam. The deviation of the retardance of the used QWP from perfect limits the maximum ellipticity angle of the pump to 43.9°. **b** Numerical simulation of patterns generated by a thin isotropic layer with *χ*^(3)^ nonlinearity when illuminated with a tightly focused laser beam. Scale bars, 2 μm

Figure 2a shows optical images of patterns at TH, obtained for decreasing angles $\gamma$ between the fast axis of the wave plate and the polarization direction of the input laser beam. This corresponds to decreasing absolute values of the ellipticity angle $\alpha$ of the pump beam entering the objective lens and the angle of the polarization ellipse inclination $\beta$. The angles are defined through Stokes parameters as $2\alpha ={\rm{asin}}\left({S}_{3}/{S}_{0}\right)$, and $2\beta ={\rm{atan}}\left({S}_{2}/{S}_{1}\right)$^46,47^. We find out that irradiating a thin a-Si film by a tightly focused pump beam with polarization close to circular, results in the generation of a TH signal with two minima in its intensity distribution (see Fig. 2a, ellipticity angles $\left|\alpha \right|={43.9}^{\circ }$). In Fig. 2, we denote the absolute value of $\alpha$, assigning a positive sign for the right-handed input and a negative sign for the left-handed input. Here we start with a maximum achievable value of $\left|\alpha \right|={43.9}^{\circ }$ (vs ${45}^{\circ }$ for a pure CP light) due to the deviation of the QWP retardance from a perfect one.

As the ellipticity angle decreases, we observe the formation of a spiral pattern with two lobes, which then transforms into a shape with one maximum at the center of the pattern. To characterize the spatial phase distribution of the generated TH signal, we also perform off-axis interference measurements, which reveal a fork-shaped pattern with a fringe difference of 2 between the top and bottom (see Suppl. Inf. I). We should note that a parasitic TH signal is generated by the setup optics; however, it does not contribute to the observed patterns, due to significant loss in the a-Si film at the TH frequency (see Suppl. Inf. H)^48^.

The experimental results can be well reproduced in the numerical simulations shown in Fig. 2b. Before describing the details of the numerical simulations in the section “Numerical simulation”, we show that the experimental results can be understood through the conservation of the TAM projection on the optical axis.

### Theoretical setting

As shown in Fig. 1, the material system is invariant under rotations along the optical axis when considered from right after the QWP up to the camera. Crucial for such invariance is the isotropy of the a-Si where the TH is generated. Symmetry then dictates that the TAM $z$-projection ($m$) on the optical axis is conserved. The precise meaning of this conservation in nonlinear interactions deserves clarification. Let us assume that the incident pump light has TAM projection ${m}_{{\rm{\omega }}}=1$^49^. Then, since three photons of the incident beam are combined in the material, the TH output must have TAM projection ${m}_{3{\rm{\omega }}}=3$. So, the conservation due to symmetry leads to tripling in this case. Such kinds of predictions can be rigorously obtained using the expressions of the angular momentum operators in tensor product spaces, as done in [see ref. ^50^, Sec. III-C] for second-harmonic generation.

However, when the input contains a superposition of more than one component of ${m}_{{\rm{\omega }}}$, e.g., $1$ and $-1$, the same conservation law implies that each possible combination of three instances of the input components will produce a TH response with ${m}_{3{\rm{\omega }}}$ equal to the sum of the three contributions.

For example, in the top row of Fig. 2, the incident beam has evolved from an almost pure content of ${m}_{{\rm{\omega }}}=1$ to a mix, in which the intensity of an additional ${m}_{{\rm{\omega }}}=-1$ component grows as the ellipticity angle of the pump decreases from $\left|\alpha \right|=43.9^\circ$ to $\left|\alpha \right|=40.8^\circ$. In the mixed case, the possible values of TAM projection for TH light are hence ${m}_{3\omega }\,\in \,\{3,1,-1,-3\}$. In our setup, components other than ${m}_{3{\rm{\omega }}}=3$ can only appear if the input pump is not a pure RCP beam. It is important to mention that the TH generation from a normally incident CP plane-wave pump is forbidden by selection rules^51^ in a film of isotropic material such as a-Si.

We highlight that the present isotropic case is the simplest one. In a general sample, both its shape and its material can have different degrees of rotational symmetry. A simple rule has been put forward to deal with the general case^52^. When a pump beam with ${m}_{\omega }$ generates the $q$-th harmonic, its final TAM projection ${m}_{q\omega }$ may be equal to any of the following values:

1 $${m}_{q\omega }=q{m}_{\omega }+{m}_{{\rm{t}}{\rm{e}}{\rm{n}}{\rm{s}}}+\nu {\mathfrak{n}},\nu \,\in \,Z$$

where the possible values of ${m}_{\text{tens}}$ are determined by the rotational properties of the nonlinear susceptibility tensor $\hat{\chi }$ of the material, and ${\mathfrak{n}}$ is the order of the rotational axis of the shape of the nanostructure, that is, the shape is invariant under rotations by angle $2\pi /{\mathfrak{n}}$. In our experiment, $q=3$, and the full rotational symmetry implies ${m}_{\text{tens}}=0$, and ${\mathfrak{n}}{\mathfrak{\to }}\infty$, $\nu =0$ in Eq. (1). When ${m}_{{\rm{\omega }}}=1$, we recover the tripling case ${m}_{3{\rm{\omega }}}=3$.

The experimental results can be understood by considering the expansion of the pump and TH beams into Bessel beams with well-defined TAM $z$-projection, and well-defined helicity. Helicity is the generalization of the circular polarization handedness of plane waves onto general Maxwell fields. Any given electromagnetic field $\left({\bf{E}},{\bf{B}}\right)$ can be decomposed into its two helicity components $\pm 1$ as^32,53,54^:

2 $${{\bf{\Lambda }}}_{\pm }=\sqrt{\frac{\epsilon }{2}}\left({\bf{E}}\pm {\text{i}}c{\bf{B}}\right)$$

where $\epsilon$ is the permittivity and $c$ the light velocity in the medium. A field of well-defined helicity is one where all its composing plane waves have the same polarization handedness. In such a case, only one of the two helical fields in Eq. (2) is different than zero. The split written in Eq. (2) is valid for general Maxwell fields, in particular, collimated fields and focused fields.

Any electromagnetic beam can be expanded into Bessel beams, but they are particularly useful for analyzing cylindrically symmetric experiments^31,32,55,56^ such as the current one. This is because both the pump and the TH beams contain very few kinds of Bessel beams with fixed TAM $z$-projection $m$, and helicity (handedness) ${\rm{\lambda }}=\pm 1$, which we will denote by $\left(m,{\rm{\lambda }}\right)$:

3 $$\left(m,{\rm{\lambda }}\right){\rm{:= }}{\int }_{0}^{{\theta }_{\max }}{\rm{d}}\theta \sin \theta {c}_{m{\rm{\lambda }}}\left(\theta \right){{\mathscr{B}}}_{m{\rm{\lambda }}}^{k\theta }({\bf{r}})$$

where $\theta ={\rm{acos}} \,({k}_{z}/k)$ is the angle of the cone that defines the Bessel beam in Fourier space, $k=\omega /c$, and ${k}_{z}$ is the projection of the wavevector onto the $z$-axis, which is the same for all the plane waves in such cone, and we assume here that ${k}_{z} > 0$. Intuitively, collimated beams only contain very small values of $\theta$ in their expansion (${\theta }_{\max }\to 0$), while focused beams feature larger ${\theta }_{\max }$. The full expression of the Bessel beams ${{\mathscr{B}}}_{m{\rm{\lambda }}}^{k\theta }\left({\bf{r}}\right)$ can be found in Suppl. Inf. B. The ${c}_{m{\rm{\lambda }}}\left(\theta \right)$ in Eq. (3) are complex coefficients, whose explicit value is not necessary to qualitatively explain the experimentally obtained images. For a given $\left(m,{\rm{\lambda }}\right)$, the phase singularities in each polarization are determined by $m$, and ${\rm{\lambda }}$ determines which polarization dominates in the collimated limit, as shown in Tab. 1. We note that the definition of a right circularly polarized (RCP, unit vector ${{\bf{e}}}_{R}$) and left circularly polarized (LCP, unit vector ${{\bf{e}}}_{L}$) field used here is opposite to the one used in^31,32,55^. Table 1 highlights the important fact that in-plane polarization and helicity are not the same thing. Beams of well-defined helicity can contain the three polarization components ${{\bf{e}}}_{R}$, ${{\bf{e}}}_{L}$, and ${{\bf{e}}}_{z}$. Such a situation is rather common. For example, a collimated circularly polarized Gaussian beam also has non-zero weights in all three components, but one of them overwhelmingly dominates over the other two. Such dominance is reduced upon focusing, which increases ${\theta }_{\max }$, and with it, the relative amplitudes of the other two components (see Table 1, top row). Even a circularly polarized plane wave, which can be seen as the exceptional case of containing a single polarization vector, will acquire some intensity on the other two polarizations upon focusing (see Suppl. Inf. D.2.1).

**Table 1:** With $\varphi ={\mathrm{atan}}\left(y,x\right)$, the first row shows the phase singularities attached to each polarization of a $\left(m,\lambda \right)$ beam of well-defined ${\text{TAM}}=m$ and helicity $\lambda =\pm 1$

	${{\bf{e}}}_{R}=\left(\hat{{\bf{x}}}+{\text{i}}\hat{{\bf{y}}}\right)/\sqrt{2}$	${{\bf{e}}}_{L}=\left(\hat{{\bf{x}}}-{\text{i}}\hat{{\bf{y}}}\right)/\sqrt{2}$	${{\bf{e}}}_{z}$
$\left(m,{\rm{\lambda }}\right)$ ${\theta }_{\max }\to 0,\left(m,1\right)$ ${\theta }_{\max }\to 0,\left(m,-1\right)$	${J}_{m-1}\exp \left({\text{i}}\left(m-1\right)\varphi \right)$ $\to {J}_{m-1}\exp \left({\text{i}}\left(m-1\right)\varphi \right)$ $\to 0$	${J}_{m+1}\exp \left({\text{i}}\left(m+1\right)\varphi \right)$ $\to 0$ $\to {J}_{m+1}\exp \left(\text{i}\left(m+1\right)\varphi \right)$	${J}_{m}\exp \left(\text{i}\mathrm{m\varphi }\right)$ $\to 0$ $\to 0$

The argument of the Bessel functions ${J}_{n}\left(\cdot \right)$ is suppressed, as it changes with $\theta$ in Eq. (3). The second and third rows show the dominant polarization component in the collimated limit (see Suppl. Inf. B)

In our case, the imperfection of the QWP implies that we always need to consider the superposition of two Gaussian beams with opposite polarization handedness. By matching their respective dominant polarizations, and requiring the presence of light in the center, Tab. 1 readily determines that the RCP input pump beam is of kind $\left(m=1,{\rm{\lambda }}=1\right)$, and the LCP input pump beam of kind $\left(m=-1,{\rm{\lambda }}=-1\right)$.

**Fig. 3:**
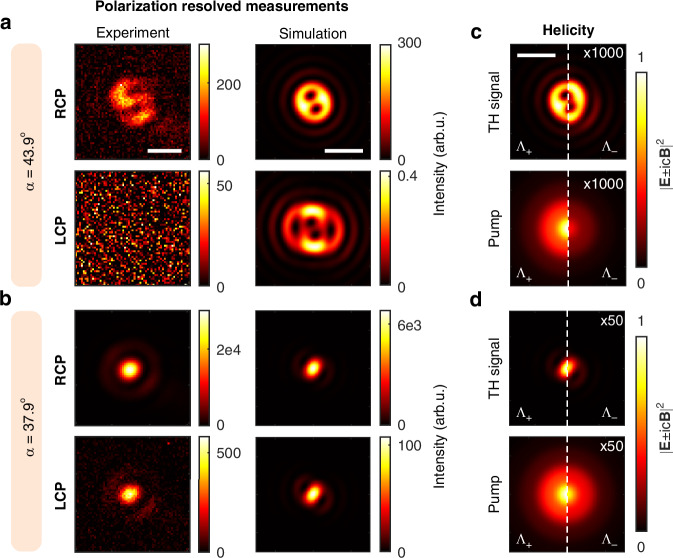
Polarization-resolved intensity and helicity of third-harmonic light. Optical images and numerical simulations of polarization resolved patterns excited with an elliptically polarized pump laser with *α* = 43.9° (**a**) and 37.9° (**b**) Numerical simulations of helicity of the pump and generated TH signal for *α* = 43.9° (**c**) and 37.9° (**d**) Electric fields were calculated on the film surface, helicity values are normalized by the corresponding maximum value for Λ_+_. Scale bars, 2 μm

The rotational symmetry will preserve the TAM $z$-projection in the sense explained above. The helicity, however, is allowed to change as long as the interaction is not symmetric under the electromagnetic duality transformation^34,54,57,58^. Duality symmetry requires that the electric and magnetic responses of the medium are of the same order, which does not occur in natural materials at optical frequencies. In non-dual symmetric systems, the efficiency of helicity conversion can vary^32^. In order to observe this, we have also conducted a polarization resolved experiment, probing the RCP and LCP of the TH signal; this has been accomplished by introducing a QWP and a polarizer on the TH beam optical path before the imaging camera (see the setup in Suppl. Inf. F). Figure 3a,b shows images of the TH signal obtained with pump polarization close to circular, $\alpha ={43.9}^{\circ }$, and deviated to $\alpha ={37.9}^{\circ }$. We see that when the pump is almost circularly polarized ($\alpha ={43.9}^{\circ }$), the TH signal is detected only when probing the signal with the same handedness as the pump, while it is not visible when probing the opposite polarization. Therefore, we may say that up to the sensitivity of the measurements, helicity is preserved in the TH generated towards the transmission direction in our system. Small helicity conversion is also observed in our numerical simulations, as can be appreciated in Fig. 3c. For our case, we assume that when three fields at the fundamental frequency combine to produce a TH field ${F}_{1}\otimes {F}_{2}\otimes {F}_{3}\to T$, the dominant helicity of the TH is determined by the prevailing helicity among the fundamental fields.

The elliptically polarized pump is composed by two kinds of $\left(m,{\rm{\lambda }}\right)$ beams: $\approx \left(1,1\right)+\varepsilon \left(-1,-1\right)$, where $\left|\varepsilon \right|$ increases as the ellipticity angle $\alpha$ decreases. Then, the allowed TH processes are:

4 $$\begin{array}{l}\left(1,1\right)\otimes \left(1,1\right)\otimes \left(1,1\right)\to \left(3,1\right)\\ \left(\mathrm{1,1}\right)\otimes \left(1,1\right)\otimes \varepsilon \left(-1,-1\right)\to \varepsilon \left(1,1\right)\\ \begin{array}{l}\left(1,1\right)\otimes \varepsilon \left(-1,-1\right)\otimes \varepsilon \left(-1,-1\right)\to {\varepsilon }^{2}\left(-1,-1\right)\\ \varepsilon \left(-1,-1\right)\otimes \varepsilon \left(-1,-1\right)\otimes \varepsilon \left(-1,-1\right)\to {\varepsilon }^{3}\left(-3,-1\right)\end{array}\end{array}$$

To 0-th order in $\varepsilon$, we obtain a $(\mathrm{3,1})$ beam, which, according to Tab. 1, features a vortex of charge 2 in its dominant ${{\bf{e}}}_{R}$ polarization: ${J}_{2}\exp \left(\text{i}2\varphi \right){{\bf{e}}}_{R}$. This contribution dominates in the top left panel of Fig. 2, where ${\rm{\varepsilon }}\approx 0.02$. The second-order phase singularity in the TH vortex generated by the film is manifested by the appearance of two additional fringes at the top of the interference pattern (see Fig. S5, Suppl. Inf. I). As $\varepsilon$ grows, other components become more visible ($\varepsilon \approx 0.07$ at $\alpha ={40.8}^{\circ }$). For example, at first order in $\varepsilon$, we obtain a $(\mathrm{1,1})$ beam: ${J}_{0}{{\bf{e}}}_{R}$, whose intensity maximum is at the center.

Importantly, for a pure (3, 1) or (1,1) beam, the intensity distribution does not depend on $\varphi$. The sequence of images seen when going from the left to the right of the top row of Fig. 2 can be qualitatively understood as the coherent linear superposition of the $\left(\mathrm{3,1}\right)$ and (1,1) components, where the latter gains relative importance as the ellipticity of the beam grows. We note that the angular dependence of the generated patterns can be retrieved solely by considering the symmetry of the system (see Suppl. Inf. D.1). The instability of optical vortices with charge higher than one^59^, which causes their split into single vortices as soon as another beam is present, can be seen from the start of the sequence. The two lobes in the main ring of the intensity patterns also match expectations: The number of lobes equals the difference between the ${m}_{3{\rm{\omega }}}$ of the two interfering beams. Finally, the bottom panel of the polarization resolved images in Fig. 3b shows a weak TH LCP component with maximum intensity in the center generated by an elliptical beam with $\alpha =37.9^\circ$. This is consistent with the $\left(-1,-1\right)$ dominant component which appears at order ${\varepsilon }^{2}$, which is a beam whose dominant polarization has a maximum at the center and exhibits no phase singularity. Order ${\varepsilon }^{2}$ is also the lowest order at which a component of changed helicity appears. We also note that low polarization purity of the input pump beam can preclude the observation of two-lobe patterns under low-NA objective focusing (see Suppl. Inf. K).

### Numerical simulation

**Fig. 4:**
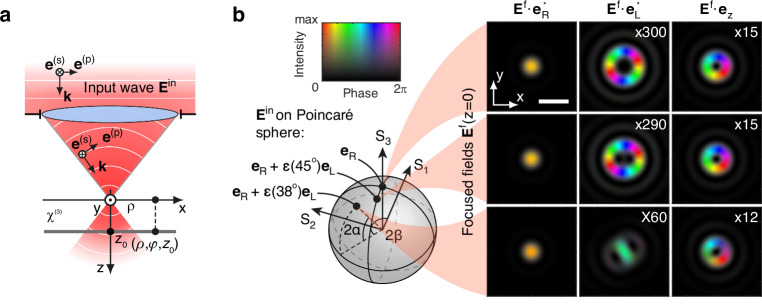
Intensity, phase and polarization properties of tightly focused light. **a** Schematic of the focusing system and the reference frame. **b** Numerically calculated intensity, ${\left|{{\bf{E}}}^{{\rm{f}}}\cdot {{\bf{e}}}_{i}^{{\boldsymbol{* }}}\right|}^{2}$, and phase, $\arg \,({{\bf{E}}}^{{\rm{f}}}\cdot {{\bf{e}}}_{i}^{\ast })$, distributions of electric fields in the focal plane (*z*_0_ = 0) of the lens (NA = 0.85) for input wave **E**^in^ ellipticity angles *α* = 45°, 43.9° and 37.9°, from top to bottom. An input RCP plane wave (located at the pole of the Poincaré sphere, *α* = 45°) undergoes transformation under focusing, resulting in a beam that includes all three polarization components: ${{\bf{e}}}_{R}$, ${{\bf{e}}}_{L}$ and ${{\bf{e}}}_{z}$. Patterns are normalized to the maximum intensity of the corresponding ${\left|{{\bf{E}}}^{{\rm{f}}}\cdot {{\bf{e}}}_{R}^{* }\right|}^{2}$ distribution. Scale bar, 2 μm

In our experiment, the size of the entrance pupil of the objective focusing the pump laser was much smaller than the diameter of the incident Gaussian beams, giving nearly uniform illumination of the entrance aperture. Therefore, we can approximate the pump beam entering the objective as a linear combination of circularly polarized plane waves:

5 $${{\bf{E}}}^{\text{in}}\propto {{\bf{e}}}_{R}+\varepsilon \left(\gamma \right){{\bf{e}}}_{L}$$

where the parameter $\varepsilon \left(\gamma \right)$ determines the contribution of the LCP wave and can be expressed through the angle $\gamma$ between the direction of oscillation of the input $y$-polarized light and the fast axis of the wave plate, and its retardance angle $\tau$ (see Suppl. Inf. E). The analytical expression of the focused electric field after the lens can

be obtained (see Eq. S16)^60,61^. Figure 4a illustrates the reference frame. In terms of $s$- and $p$-polarized plane waves, with polarization vectors determined as follows^62^:

6 $${{\bf{e}}}^{(\text{s})}=\frac{1}{{k}_{\rho }}\left[{k}_{y},-{k}_{x},0\right]$$

7 $${{\bf{e}}}^{(\text{p})}=\frac{1}{{k}_{\rho }k}\left[{k}_{z}{k}_{x},{k}_{z}{k}_{y},-{k}_{\rho }^{2}\right]$$

the focused electric field is given by:

8 $${{\bf{E}}}^{\text{f}}\left(\rho ,\varphi ,z\right)=A\mathop{\int }\limits_{{k}_{x}^{2}+{k}_{y}^{2} < {k}_{\rho 0}^{2}}\frac{g\left({k}_{z}\right)}{{k}_{z}}\left[\left({{\bf{E}}}^{\text{in}}\cdot {{\bf{e}}}^{(\text{s})}\right){{\bf{e}}}^{(\text{s})}+\left({{\bf{E}}}^{\text{in}}\cdot \left({{\bf{e}}}_{z}\times {{\bf{e}}}^{(\text{s})}\right)\right){{\bf{e}}}^{(\text{p})}\right]{e}^{\text{i}{\bf{k}}\cdot {\bf{r}}}{\rm{d}}{k}_{x}{\rm{d}}{k}_{y}$$

where $A$ is the normalization constant, $g\left({k}_{{\rm{z}}}\right)$ is the apodization factor ($g\left({k}_{z}\right)=\sqrt{{k}_{z}/k}$ for an aplanatic focusing system^60,61^), $k=\omega /c$, ${k}_{\rho 0}$ is the maximum value of the in-plane projection of the wavevector and it is equal to $k{\text{NA}},{k}_{q}({\text{with}}\,q=x,y,z)$ are the Cartesian components of the wavevector, and ${{\bf{E}}}^{\text{in}}$ is the electric field of the input plane wave. The field in the form of an integral in Eq. (8) can be retrieved using efficient computational algorithms^63^.

Figure 4b shows the intensity and phase distributions of focused plane waves with $\alpha =45^\circ ,43.9^\circ$ and $37.9^\circ$ projected onto ${{\bf{e}}}_{R}$, ${{\bf{e}}}_{L}$ and ${{\bf{e}}}_{z}$ basis vectors. For a pure RCP plane wave ($\alpha ={45}^{\circ }$), the intensity of the electric field component with the same polarization as the input, ${{\bf{E}}}^{\text{f}}\cdot {{\bf{e}}}_{R}^{* }$, is maximal and its phase does not exhibit a helical profile. A helical phase is present for the oppositely polarized, ${{\bf{E}}}^{\text{f}}\cdot {{\bf{e}}}_{L}^{* }$, and longitudinal components, ${{\bf{E}}}^{\text{f}}\cdot {{\bf{e}}}_{z}$, as expected. The input RCP plane wave can be seen as a (1,1) beam in Tab. 1 with ${\theta }_{\max }\to 0$, where the only significant polarization component is the one which features a ${J}_{0}$ Bessel function without a phase singularity. The other two polarizations feature Bessel functions of higher order, whose amplitude is much suppressed by their vanishing $k\sin \theta \rho$ argument, and the $\theta$-dependent factors multiplying the Bessel functions.

Upon focusing, which preserves both $m$ and ${\rm{\lambda }}$^54^, the value of ${\theta }_{\max }$ increases, and with it the relative strength of the other two polarizations, with their corresponding phase singularities: ${J}_{2}\exp \left(\text{i}2\varphi \right){{\bf{e}}}_{L}$, and ${J}_{1}\exp \left(\text{i}\varphi \right){{\bf{e}}}_{z}$.

When using an imperfect quarter-wave plate, the maximum absolute value of ellipticity angle is achieved at $\gamma ={45}^{\circ }$, and, in our case, is equal to $\alpha =43.9^\circ$. Therefore, the point representing the polarization state of the input radiation shifts from the pole of the Poincaré sphere. At the same time, the oppositely polarized component of the focused field, ${{\bf{E}}}^{\text{f}}\cdot {{\bf{e}}}_{L}^{* }$, exhibits some asymmetry and an increase in intensity (see Fig. 4b, middle panel). Further reduction of the ellipticity angle, achieved by deviating the wave plate to $\gamma =38^\circ$ ($\alpha =37.9^\circ$), leads to a further increase in the intensity of ${{\bf{E}}}^{\text{f}}\cdot {{\bf{e}}}_{L}^{* }$ component and a significant change in its distribution, with a maximum emerging at the center (see Fig. 4b, bottom panel). We also note that the fit of the numerically calculated TH field with the set of terms given in Eq. 4 shows good agreement, supporting the validity of the theoretical consideration (see Suppl. Inf. J).

In the numerical model, we approximate the a-Si film by an infinitely thin nonlinear layer placed in air at $z={z}_{0}$. The nonlinear polarization density for the TH generation in the nonlinear layer is written as^45^:

9 $${{\bf{P}}}^{3\omega }\left(\rho ,\varphi \right)={\hat{\chi}}^{(3)}:\left({{\bf{E}}}^{\omega }\left(\rho ,\varphi \right)\otimes {{\bf{E}}}^{\omega }\left(\rho ,\varphi \right)\otimes {{\bf{E}}}^{\omega }\left(\rho ,\varphi \right)\right)$$

where ${{\bf{E}}}^{\omega }\left(\rho ,\varphi \right)={{\bf{E}}}^{\text{f}}\left(\rho ,\varphi ,{z}_{0}\right)$ is determined from Eq. (8). The components of ${\hat{\chi}}^{(3)}$ can be found in Suppl. Inf. C. The nonlinear polarization source generates electric fields that are calculated using the Green’s function approach^64^. We find that misalignment along the propagation axis between the pump beam focus position and the film plane affects the shape of the TH field. In order to achieve the best match between the simulation and the experimental data we introduce a shift in the position of the waist of the pump beam by *z*_0_ = −2.055 μm. Figure 2b shows the TH patterns calculated numerically. In the model we neglected the presence of the substrate, because it does not qualitatively affect the results (see Suppl. Inf. L). The numerically calculated patterns were averaged over the focal depth of the collecting objective lens (NA = 0.4).

### Helicity change in the TH simulation

Since focusing preserves ${\rm{\lambda }}$, the pump electric field produced by a focused RCP plane wave can be represented as a sum of plane waves of the same handedness, by construction, giving the ratio $\int {\left|{{\bf{\Lambda }}}_{-}\right|}^{2}{\text{d}}S/\int {\left|{{\bf{\Lambda }}}_{+}\right|}^{2}{\rm{d}}S=0$, where the surface integrals are performed in the plane $z={z}_{0}$. In our model, the duality symmetry inherent in vacuum is broken by the nonlinear interaction in an obvious way: The absence of magnetic terms in Eq. (9) negates the electric-magnetic equivalence required by duality. For the TH generated by a focused RCP plane wave we obtain numerically $\int {\left|{{\bf{\Lambda }}}_{-}\right|}^{2}{\rm{d}}S/\int {\left|{{\bf{\Lambda }}}_{+}\right|}^{2}{\rm{d}}S=1.43\times {10}^{-4}$, which implies a rather small helicity conversion factor.

Figure 3c shows the helicity of a pump beam with an ellipticity angle $\alpha ={43.9}^{\circ }$ and the generated TH signal. In this case, for the pump $\int |{{\bf{\Lambda }}}_{-}{|}^{2}{\rm{d}}S/\int |{{\bf{\Lambda }}}_{+}{|}^{2}{\rm{d}}S=3.88\times {10}^{-4}$ becomes non-zero, due to the presence of an opposite polarized component at the input ($\varepsilon \,\ne \,0$ in Eq. (5)). Bottom panel of Fig. 3c shows the helicity of the corresponding nonlinear signal. The change of helicity is again small, and the relative strength $\int |{{\bf{\Lambda }}}_{-}{|}^{2}{\rm{d}}S/\int |{{\bf{\Lambda }}}_{+}|^{2}{\rm{d}}S=1.1\times {10}^{-3}$ prevents one from experimentally detecting a signal in the bottom-left panel of Fig. 3a. It should be noted that the relatively small helicity changes have been obtained for the TH generated towards the transmission direction, and they do not rule out a much larger helicity conversion in reflection.

## Discussion

In this work, we explored light structuring via nonlinear total angular momentum addition in thin films of amorphous silicon. Through both experimental measurements and numerical simulations, we demonstrated the capability of thin layers of isotropic material to enable tripling of the TAM projection under tightly focused laser excitation. The suggested numerical model provides results that are in strong qualitative and quantitative agreement with the experimental data. Our experimental measurements reveal that the change in the helicity of the generated TH signal, inherent to nonlinear interactions, is undetectable within our experimental setup. This finding aligns with numerical simulations, which predict a helicity conversion of less than 0.05% for a circularly polarized focused pump. The negligible helicity conversion in nonlinear generation under tightly focused fields supports the use of TAM and helicity as key parameters for analyzing experimental data. Our theoretical framework, based on Bessel beams of well-defined helicity, provides a clear and powerful approach for accurate analysis of experiments involving non-paraxial fields and can be applied to the design of new optical devices that exploit symmetry for enhanced control of light structure. Nonlinear flat thin layers offer exciting functionalities for light shaping applications, combining ultra-compact design with relaxed phase-matching conditions. Additionally, the demonstrated high sensitivity of the shape of the generated TH patterns to the purity of excitation polarization offers a promising method for circularly polarized light detection^65^. This study advances the understanding of nonlinear light–matter interactions using flat isotropic media to achieve sophisticated light tailoring and contributes to the ongoing development of nonlinear flat optics. Several studies have addressed structured harmonic generation using paraxial concepts referred to as OAM and SAM^66–70^. We do not aim to provide an exhaustive overview of all works related to this broad topic. Although TAM projection conservation is often mentioned in these studies, the focus is typically placed on paraxial quantities, which makes the interpretation less natural. In fact, the final results in all such cases can be readily derived from the conservation of the TAM projection. Furthermore, we emphasize the importance of a careful treatment of material structure; for instance, in ref. ^69^, the tensor-induced ${m}_{\text{tens}}$ in second-harmonic generation is restricted to $\pm 3$ (in case of material ${D}_{3h}$ symmetry) rather than arbitrary integer values.

We would like to stress once again that our approach, which involves considering the total angular momentum projection and helicity, is symmetry-based and universal, as it does not rely on the paraxial approximation and allows all observed effects to be explained without introducing additional entities and/or approximations.

## Methods

A thin layer (1 μm) of amorphous silicon was deposited by means of electron beam (e-beam) evaporation (deposition rate 0.4 nm/s, base vacuum $2\times {10}^{-7}$ mbar) from a target made of Si pellets 99.99% on a fused silica (SiO_2_) substrate previously cleaned using acidic piranha solution (H_2_SO_4_:H_2_O_2_).

As a pump laser source, we used the emission of an optical parametric amplifier (Coherent Opera-F) coupled with a femtosecond laser system (Coherent MONACO), operating at 1035 nm with the pulse duration of $\approx$300 fs and 500 kHz repetition rate. The pump light at a wavelength of 1500 nm was focused on the sample with an Olympus LCPlan N 100X/0.85 IR objective, the TH signal was collected by an Olympus LMPlanFL N 20x/0.40 objective and filtered by a short pass filter (Thorlabs FESH0950). To transform the polarization of a linearly polarized input light, we used an achromatic quarter-wave plate (Thorlabs AQWP10M-1600) with a retardance of 0.2437 at 1500 nm. To inspect the polarization of the generated TH, we specifically employed a quarter-wave plate (Thorlabs AQWP10M-580) and a polarizer (Thorlabs GL10-A).

## Supplementary information

Supplementary Information: Light structuring via nonlinear total angular momentum addition with flat optics

## Data Availability

Data underlying the results in this paper may be obtained from the authors upon reasonable request.
