# Supplementary material for: Light structuring via nonlinear total angular momentum addition with flat optics
Source: Light Sci Appl. 2025 Nov 12;14:381. doi: 10.1038/s41377-025-02004-8 (PMC12612144; doi:10.1038/s41377-025-02004-8)
Supplement: Supplementary file 1 — Supplementary Information: Light structuring via nonlinear total angular momentum addition with flat optics [file 41377_2025_2004_MOESM1_ESM.pdf]

Supplementary Information: Light structuring via nonlinear total angular momentum addition with flat optics

Evgenii Menshikov*[1], Paolo Franceschini†[1,2], Kristina Frizyuk‡[1], Ivan Fernandez-Corbaton§[3], Andrea Tognazzi¶[2,4], Alfonso Carmelo Cino‖[4], Denis Garoli**[5], Mihail Petrov††[6], Domenico de Ceglia‡‡[1, 2], and Costantino De Angelis§§[1, 2]

[1]Department of Information Engineering, University of Brescia, Via Branze, 38, Brescia 25123, Italy
[2]National Institute of Optics-National Research Council, Via Branze, 45, Brescia, 25123, Italy
[3]Karlsruhe Institute of Technology, Kaiserstrasse, 12, Karlsruhe, 76131, Germany
[4]Department of Engineering, University of Palermo, Viale delle Scienze, 9, Palermo, 90128, Italy
[5]Dipartimento di Scienze e Metodi dell'Ingegneria, Università degli Studi di Modena e Reggio Emilia, Via Amendola, 2, Reggio Emilia, 43122, Italy
[6]New Uzbekistan University, Mirzo Ulugbek, Movarounnahr, 1, Tashkent, Uzbekistan

Abstract

The Supplementary Information provides detailed theoretical and experimental support for the main article. It consists of the following sections:

Contents

A Definitions **2**

B Bessel Beams of well-defined helicity **2**

C $\chi^{(3)}$ of isotropic nonlinear medium **3**

D Theory of the TAM projection tripling in amorphous silicon **3**
- D.1 Angular dependence 3
- D.2 Radial dependence 3
  - D.2.1 On particular form of radial dependence in our case 4
- D.3 Polarization and electric field distribution at TH generated by a focused plane wave . 4

E Expression for $\varepsilon(\gamma)$ **5**

F Schematic of the optical setup **6**

G Polarization resolved measurements of TH signal **6**

H Background TH generation by the optics of the setup **6**

I Off-axis interferometric measurements **7**

J Quantitative analysis of the TAM composition **8**

K TH generation under excitation through low-NA objective **11**

L Numerical simulation of TH generation with and without the substrate **11**

M THG conversion efficiency **12**

*evgenii.menshikov@unibs.it
†paolo.franceschini@unibs.it
‡frzyuk@gmail.com
§ivan.fernandez-corbaton@kit.edu
¶andrea.tognazzi@unipa.it
‖alfonsocarmelo.cino@unipa.it
**denis.garoli@unimore.it
††trisha.petrov@gmail.com
‡‡Corresponding author: domenico.deceglia@unibs.it, +39 0303715590
§§costantino.deangelis@unibs.it

A Definitions

Total angular momentum of a field is a number $J \in \mathbb{N}_0$, labeling the irreducible representation of the group SO(3) with dimension $2J+1$ and characterizing the field's behavior under rotations in 3D space [1, 11.4.2], [2, "Eigenfunctions of the Photon Angular Momentum Operator"].

There exist $2J+1$ eigenfunctions of the total angular momentum operator $\hat{J}^2$ with eigenvalue $J(J+1)$, which transform through each other according to the J-th representation of the rotation group SO(3). One of the simplest examples are spherical functions Y_{Jm} [3, 8.4]. Another examples are electric or magnetic vector spherical harmonics.

Total angular momentum projection of a field is a number $m \in \mathbb{Z}$, characterizing the field's behavior under rotations around the z-axis. The function characterized by TAM projection m does not change its shape and acquires a phase $e^{\mathrm{i}m\alpha}$ under such rotations, where α is the angle of rotation.

Note that one can also define the TAM projection through the representations of the symmetry group of rotations of a cone (all rotations around a single axis), and the field can possess a well-defined m even if it consists of a sum of all possible $J \geq |m|$.

Helicity of a field

As an operator, helicity Λ is the projection of the angular momentum vector $\mathbf{J}$ onto the direction of the linear momentum vector $\mathbf{P}$,

$$\Lambda = \frac{\mathbf{J}\cdot\mathbf{P}}{|\mathbf{P}|}. \tag{S1}$$

Equation (S1) is the most general form of the helicity operator, which is valid for many particles and fields, such as electrons and gravitational waves. For Maxwell fields, helicity describes the sense of screw in light: The circular polarization handedness.

Helicity characterizes the behavior of electromagnetic fields under duality transformations. Fields with helicity λ just acquire a phase $e^{-\mathrm{i}\lambda\vartheta}$ under such transformations, where ϑ is the "angle", characterizing the duality transformation [4].

In the context of representation theory, the eigenvalues of the helicity operator, $\lambda = \pm 1$ in case of photons, label the irreps of the little group of the Poincaré group E(2), for massless particles, [1, 10.4.4].

B Bessel Beams of well-defined helicity

A Bessel beam can be constructed as a weighted integral sum of all the plane waves with the same frequency ω, z-component of the wavevector k_z, and polarization. Different polarization basis can be used. When using the linear polarization basis the plane waves can be selected to be either all s-polarized or all p-polarized [5], whereas when circular polarization handedness are used [6, 7], all the plane waves have either positive or negative helicity. The wavevectors of all the plane waves in the integral determine a conical surface in k-space, the angle of the cone is equal to $\theta = \mathrm{acos}\,(k_z/k)$. The weights in the integral are selected so that, upon rotation around the z axis, the beam maintains its form and picks up a phase equal to $\exp(-\mathrm{i}m\varphi)$. That is, the Bessel beams are eigenstates of the TAM projection operator with eigenvalue m. If the handedness basis is chosen for the plane waves, they are also eigenstates of the helicity operator with eigenvalues $\lambda = 1$ or $\lambda = -1$. For a fixed wavenumber $k = \omega/c$, the explicit forms of the Bessel beams $\mathcal{B}^{k\theta}_{m\lambda}(\mathbf{r})$ in cylindrical coordinates $[\rho = \sqrt{x^2+y^2}, \varphi = \mathrm{atan}(y,x), z]$ are [4, Eq. (2.82)], [6, Eq. (11)]:

$$\begin{aligned}
&\mathcal{B}^{k\theta}_{m-}(\rho,\varphi,z) = \\
&= \sqrt{\frac{k|\sin\theta|}{2\pi}}\mathrm{i}^m \exp(\mathrm{i}(k\cos\theta z + m\varphi))\cdot \\
&\cdot\Bigg(\frac{\mathrm{i}}{\sqrt{2}}\left[(1+\cos\theta)\, J_{m+1}(k|\sin\theta|\,\rho)\exp(\mathrm{i}\varphi)\mathbf{e}_L + \right.\\
&\left. + (1-\cos\theta)\, J_{m-1}(k|\sin\theta|\,\rho)\exp(-\mathrm{i}\varphi)\mathbf{e}_R\right] \\
&- \frac{k|\sin\theta|}{k} J_m(k|\sin\theta|\,\rho)\mathbf{e}_z\Bigg) \\
&\mathcal{B}^{k\theta}_{m+}(\rho,\varphi,z) = \\
&= \sqrt{\frac{k|\sin\theta|}{2\pi}}\mathrm{i}^m \exp(\mathrm{i}(k\cos\theta z + m\varphi))\cdot \\
&\cdot\Bigg(\frac{\mathrm{i}}{\sqrt{2}}\left[(1-\cos\theta)\, J_{m+1}(k|\sin\theta|\,\rho)\exp(\mathrm{i}\varphi)\mathbf{e}_L + \right.\\
&\left. + (1+\cos\theta)\, J_{m-1}(k|\sin\theta|\,\rho)\exp(-\mathrm{i}\varphi)\mathbf{e}_R\right] \\
&+ \frac{k|\sin\theta|}{k} J_m(k|\sin\theta|\,\rho)\mathbf{e}_z\Bigg).
\end{aligned} \tag{S2}$$

where $J_n(\cdot)$ are Bessel functions, and the polarization vectors $[\mathbf{e}_L, \mathbf{e}_R, \mathbf{e}_z]$ are defined in Tab. 1.

The approximated forms of collimated Bessel beams can be obtained by taking the limit of small $|\theta|$: $\sin\theta \to \theta$, $\cos\theta \to 1$, and then keeping the polarization with the largest coefficient multiplying the phase factors and Bessel functions. For positive k_z/k ($0 \leq \theta \leq \pi/2$) we obtain:

$$\begin{aligned}
&\mathcal{B}^{k\theta\to 0}_{m-}(\rho,\varphi,z) \to \\
&\sqrt{\frac{k|\theta|}{\pi}}\exp(\mathrm{i}kz)\mathrm{i}^{m+1} J_{m+1}(k|\theta|\,\rho)\exp(\mathrm{i}\varphi(m+1))\mathbf{e}_L \\
&\mathcal{B}^{k\theta\to 0}_{m+}(\rho,\varphi,z) \to \\
&\sqrt{\frac{k|\theta|}{\pi}}\exp(\mathrm{i}kz)\mathrm{i}^{m+1} J_{m-1}(k|\theta|\,\rho)\exp(\mathrm{i}\varphi(m-1))\mathbf{e}_R
\end{aligned} \tag{S3}$$

C $\chi^{(3)}$ of isotropic nonlinear medium

In Cartesian coordinates, the $\chi^{(3)}_{ijkl}$ tensor of isotropic medium has the following form [8]:

$$\begin{aligned}
&\chi^{(3)}_{xxxx} = \chi^{(3)}_{yyyy} = \chi^{(3)}_{zzzz},\\
&\chi^{(3)}_{xxyy} = \chi^{(3)}_{yyzz} = \chi^{(3)}_{zzxx} = \chi^{(3)}_{zzyy} = \chi^{(3)}_{yyzz} = \chi^{(3)}_{xxzz},\\
&\chi^{(3)}_{xzxz} = \chi^{(3)}_{xyxy} = \chi^{(3)}_{yzyz} = \chi^{(3)}_{yxyx} = \chi^{(3)}_{zyzy} = \chi^{(3)}_{zxzx},\\
&\chi^{(3)}_{xyyx} = \chi^{(3)}_{xzzx} = \chi^{(3)}_{yxxy} = \chi^{(3)}_{yzzy} = \chi^{(3)}_{zyyz} = \chi^{(3)}_{zxxz}.,\\
&\chi^{(3)}_{xxxx} = \chi^{(3)}_{xxyy} + \chi^{(3)}_{xyxy} + \chi^{(3)}_{xyyx}.
\end{aligned} \tag{S4}$$

Let us provide the components with values 12, 4, 4, 4 for $\chi^{(3)}_{xxxx}$, $\chi^{(3)}_{xxyy}$, $\chi^{(3)}_{xyxy}$ and $\chi^{(3)}_{xyyx}$, respectively. Then, in cylindrical coordinates we obtain [9]:

$$\begin{aligned}
&\chi^{(3)}_{zzzz} = \chi^{(3)}_{\rho\rho\rho\rho} = \chi^{(3)}_{\varphi\varphi\varphi\varphi} = 12,\\
&\chi^{(3)}_{\rho\rho\varphi\varphi} = \chi^{(3)}_{\rho\varphi\rho\varphi} = \chi^{(3)}_{\rho\varphi\varphi\rho} = \chi^{(3)}_{\varphi\rho\rho\varphi} = \chi^{(3)}_{\varphi\varphi\rho\rho} = \chi^{(3)}_{\varphi\rho\varphi\rho} =\\
&=\chi^{(3)}_{\varphi z\varphi z} = \chi^{(3)}_{\varphi zz\varphi} = \chi^{(3)}_{z\varphi z\varphi} = \chi^{(3)}_{z\varphi\varphi z} = \chi^{(3)}_{\varphi\varphi zz} = \chi^{(3)}_{zz\varphi\varphi} =\\
&=\chi^{(3)}_{z\rho z\rho} = \chi^{(3)}_{\rho zz\rho} = \chi^{(3)}_{\rho z\rho z} = \chi^{(3)}_{zz\rho\rho} = \chi^{(3)}_{z\rho\rho z} = \chi^{(3)}_{\rho\rho zz} =\\
&=4.
\end{aligned} \tag{S5}$$

One can see that all the components do not depend on φ, from which it follows that $m_{\text{tens}} = 0$ (also by comparison with tensors for the lattices of another symmetry, given, e.g. in [9]).

D Theory of the TAM projection tripling in amorphous silicon

D.1 Angular dependence

Let us provide the theoretical description of the observed patterns using an alternative approach. Let us note that, in principle, we could use not only Bessel beams but also any beams with well-defined helicity and TAM projection, or even multipoles. This approach does not rely on a specific choice of basis. In this section, we are only interested in azimuthal dependence of all functions, more precisely, in their symmetry behavior under rotations around the propagation (z-) axis.

In cylindrical coordinates (ρ, φ, z), a circularly polarized plane wave can be written as [10]

$$\mathbf{E}^{\text{cp}} \propto (\mathbf{e}_\rho \pm \mathrm{i}\mathbf{e}_\varphi)e^{\pm\mathrm{i}\varphi}, \tag{S6}$$

where $\pm$ corresponds to the right and left handedness, respectively. The expression in Eq. (S6) has been chosen since the unit basis vectors of the cylindrical coordinate system do not change under rotation, and the exponential multiplier stands for the whole behavior. Indeed, under rotation by an angle β we just have $\varphi \to \varphi - \beta$. If the pump beam has arbitrary radial dependence and right handedness, the electric field takes the form $\mathbf{E}^\omega(\rho,\varphi) = \tilde{\mathbf{E}}^\omega(\rho)e^{\mathrm{i}\varphi}$, thus from Eq. (9) for the nonlinear polarization, since $m_{\text{tens}} = 0$, we have:

$$\mathbf{P}^{3\omega}_0(\rho,\varphi) \propto \tilde{\mathbf{P}}^{3\omega}_0(\rho)e^{3\mathrm{i}\varphi}, \tag{S7}$$

where $\mathbf{P}^{3\omega}_0(\rho)$ does not depend on φ, i.e. it is invariant under rotations. The intensity distribution, produced by this source, will possess the same symmetry behavior. However, if we the helicity of the pump is not pure, i.e. there is a contribution with the opposite handedness (opposite TAM projection), $\varepsilon e^{-\mathrm{i}\varphi}$, we will obtain additional terms

$$\mathbf{P}^{3\omega}_1(\rho,\varphi) \propto \varepsilon\tilde{\mathbf{P}}^{3\omega}_1(\rho)e^{1\mathrm{i}\varphi} \tag{S8}$$

$$\mathbf{P}^{3\omega}_2(\rho,\varphi) \propto \varepsilon^2\tilde{\mathbf{P}}^{3\omega}_2(\rho)e^{-1\mathrm{i}\varphi} \tag{S9}$$

$$\mathbf{P}^{3\omega}_3(\rho,\varphi) \propto \varepsilon^3\tilde{\mathbf{P}}^{3\omega}_3(\rho)e^{-3\mathrm{i}\varphi}. \tag{S10}$$

Now we consider the angular dependence of the total TH intensity. Taking into account only the fields produced by the first two terms, i.e. $\mathbf{P}^{3\omega}_0(\rho,\varphi)$ and $\mathbf{P}^{3\omega}_1(\rho,\varphi)$, and neglecting the terms of higher order, we obtain

$$I^{3\omega} \propto |(\mathbf{a}(\rho)e^{3\mathrm{i}\varphi} + \mathbf{b}(\rho)\varepsilon e^{1\mathrm{i}\varphi})|^2 \propto c(\rho) + d(\rho)\cos(2\varphi). \tag{S11}$$

where the vector functions $\mathbf{a}(\rho)$ and $\mathbf{b}(\rho)$ characterize the radial dependence of polarization. Equation (S11) indicates that, in general, the TH signal generated by a non-pure pump represents a two-lobe pattern (see Fig. 2). Radial dependence, described by the functions $\mathbf{a}, \mathbf{b}, c, d$ will be discussed in more detail in Section D.2.

D.2 Radial dependence

Now we focus on the radial dependence of the formed patterns. On the sample plane, one may decompose the incident wave into the terms with right, left, and longitudinal polarization. To this purpose, the following terms can be introduced

$$\frac{1}{\sqrt{2}}(\hat{\mathbf{x}} + \mathrm{i}\hat{\mathbf{y}}) = \frac{1}{\sqrt{2}}(\mathbf{e}_\rho + \mathrm{i}\mathbf{e}_\varphi)e^{\mathrm{i}\varphi} = \mathbf{e}_R \tag{S12}$$

$$\frac{1}{\sqrt{2}}(\hat{\mathbf{x}} - \mathrm{i}\hat{\mathbf{y}}) = \frac{1}{\sqrt{2}}(\mathbf{e}_\rho - \mathrm{i}\mathbf{e}_\varphi)e^{-\mathrm{i}\varphi} = \mathbf{e}_L \tag{S13}$$

$$\mathbf{e}_z = \mathbf{e}_z \tag{S14}$$

Because of rotational symmetry of the focusing system, the focused electric field should contain only terms possessing the same m, as the input electric field. Thus, from Eqs. (S12)-(S14) and assuming that they can have arbitrary radial dependence, we can deduce the form for a focused field with $m = 1$ in the most general case:

$$\mathbf{E}^{\text{f}}(r,\varphi,z) = f_0\mathbf{e}_R + f_2e^{2\mathrm{i}\varphi}\mathbf{e}_L + f_1e^{\mathrm{i}\varphi}\mathbf{e}_z. \tag{S15}$$

The exponential terms in Eq. (S15) appear to save the rotational symmetry. Therefore, it follows that $f_0(\rho, z)$ will be non-zero in the center of the distribution ($\rho = 0$), while $f_1(\rho, z)$ and $f_2(\rho, z)$ should vanish, because the angular part has a phase singularity.

D.2.1 On particular form of radial dependence in our case

For an RCP plane wave at the input, the focused field can be expressed as [11, 12]:

$$\mathbf{E}^{\mathrm{f}}(\rho,\varphi,z) \propto \frac{1}{\sqrt{2}}(I_0\mathbf{e}_R + I_2 e^{2\mathrm{i}\varphi}\mathbf{e}_L) - \mathrm{i}I_1 e^{\mathrm{i}\varphi}\mathbf{e}_z, \quad \text{(S16)}$$

where (ρ, φ, z) are the coordinates of the observation point in a cylindrical coordinate system (see Fig. 4a) and the functions $I_i(\rho, z)$, with $i = 0, 1, 2$, are expressed in integral form and explicitly given by:

$$I_0(\rho,z) = \int_0^{\theta_{\max}} g(\theta)\sin\theta(1+\cos\theta)\cdot \cdot J_0(k\rho\sin\theta)\exp(\mathrm{i}kz\cos\theta)\mathrm{d}\theta \quad \text{(S17)}$$

$$I_1(\rho,z) = \int_0^{\theta_{\max}} g(\theta)\sin^2(\theta)\cdot \cdot J_1(k\rho\sin\theta)\exp(\mathrm{i}kz\cos\theta)\mathrm{d}\theta \quad \text{(S18)}$$

$$I_2(\rho,z) = \int_0^{\theta_{\max}} g(\theta)\sin\theta(1-\cos\theta)\cdot \cdot J_2(k\rho\sin\theta)\exp(\mathrm{i}kz\cos\theta)\mathrm{d}\theta \quad \text{(S19)}$$

with $g(\theta)$ being the apodization factor, which for an aplanatic lens is equal to $\sqrt{\cos(\theta)}$, and $\theta_{\max} = \mathrm{asin(NA)}$, where NA is the objective lens numerical aperture, $k = \omega/c$.

D.3 Polarization and electric field distribution at TH generated by a focused plane wave

From Eq. (9) and taking into account the intrinsic permutation symmetry [8] for components of the polarization field induced in a thin isotropic layer at 3ω, we have:

$$\begin{aligned} P_x^{3\omega} &\propto E_xE_xE_x + E_xE_yE_y + E_xE_zE_z \\ P_y^{3\omega} &\propto E_yE_yE_y + E_yE_xE_x + E_yE_zE_z \\ P_z^{3\omega} &\propto E_zE_zE_z + E_zE_yE_y + E_zE_xE_x \end{aligned} \quad \text{(S20)}$$

Therefore, the components of the polarization induced by the focused RCP plane wave in Eq. (S15) are given by:

$$\begin{aligned} P_x^{3\omega} &\propto (4f_2^2f_0 + f_2f_1^2)e^{4\mathrm{i}\varphi} + (4f_2f_0^2 + f_0f_1^2)e^{2\mathrm{i}\varphi} \\ P_y^{3\omega} &\propto -\mathrm{i}(4f_2^2f_0 + f_2f_1^2)e^{4\mathrm{i}\varphi} + \mathrm{i}(4f_2f_0^2 + f_0f_1^2)e^{2\mathrm{i}\varphi} \\ P_z^{3\omega} &\propto (f_1^3 + 4f_2f_0f_1)e^{3\mathrm{i}\varphi} \end{aligned} \quad \text{(S21)}$$

Taking into account Eqs. (S12)-(S14), the polarization at 3ω takes the form:

$$\begin{aligned} \mathbf{P}^{3\omega}(\rho,\varphi,z) \propto\; & (4f_2f_0^2 + f_0f_1^2)e^{2\mathrm{i}\varphi}\mathbf{e}_R + \\ & + (4f_2^2f_0 + f_2f_1^2)e^{4\mathrm{i}\varphi}\mathbf{e}_L + \\ & + (4f_0f_2f_1 + f_1^3)e^{3\mathrm{i}\varphi}\mathbf{e}_z, \end{aligned} \quad \text{(S22)}$$

which can be rewritten as:

$$\mathbf{P}^{3\omega}(\rho,\varphi,z) = \tilde{\mathbf{P}}^{3\omega}(\rho,z)e^{3\mathrm{i}\varphi}. \quad \text{(S23)}$$

At this stage it is worthy to note that the presence of exponential multiplier in Eq. (S23) is consistent with prediction of the general theory, confirming the tripling of total orbital momentum projection. On the other hand, the polarization induced by an RCP plane wave with LCP contribution proportional to ε, is given as follows:

$$\begin{aligned} \mathbf{P}^{3\omega}(\rho,\varphi,z) \propto\; & g_0e^{2\mathrm{i}\varphi}\mathbf{e}_R + g_2e^{4\mathrm{i}\varphi}\mathbf{e}_L + g_1e^{3\mathrm{i}\varphi}\mathbf{e}_z + \\ & + \varepsilon(h_0\mathbf{e}_R + h_2e^{2\mathrm{i}\varphi}\mathbf{e}_L + h_1e^{\mathrm{i}\varphi}\mathbf{e}_z) + \\ & + \varepsilon^2(h_2e^{-2\mathrm{i}\varphi}\mathbf{e}_R + h_0\mathbf{e}_L + h_1e^{-\mathrm{i}\varphi}\mathbf{e}_z) + \\ & + \varepsilon^3(g_2e^{-4\mathrm{i}\varphi}\mathbf{e}_R + g_0e^{-2\mathrm{i}\varphi}\mathbf{e}_L + g_1e^{-3\mathrm{i}\varphi}\mathbf{e}_z) \end{aligned} \quad \text{(S24)}$$

where we introduced $g_0 = 4f_2f_0^2 + f_0f_1^2$, $g_1 = 4f_2f_0f_1 + f_1^3$, $g_2 = 4f_2^2f_0 + f_2f_1^2$, $h_0 = 4f_0^3 + 8f_2^2f_0 + 2f_0f_1^2 + f_2f_1^2$, $h_1 = 4f_2f_0f_1 + 3f_1^3 + 4f_2^2f_1 + 4f_0^2f_1$ and $h_2 = 4f_2^3 + 8f_2f_0^2 + 2f_2f_1^2 + f_0f_1^2$.

One can note that polarization induced by a non-pure pump field can not be represented in the form of equality (S23), since it contains terms with $m_{3\omega} \in \{3, 1, -1, -3\}$, as also shown in Eq. (4).

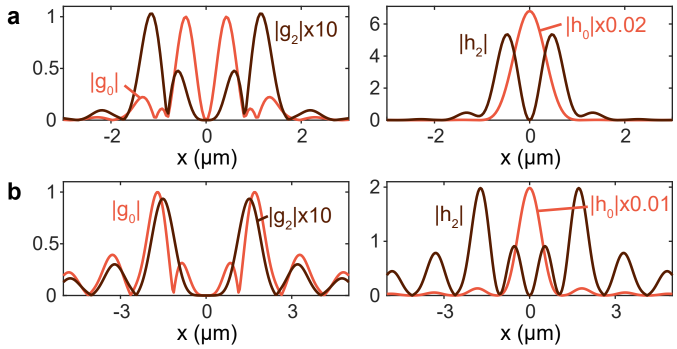

Figure S1: Calculated normalized components of polarization $\mathbf{P}^{3\omega}(z_0 = 0)$ with pump being (a) a focused plane wave (NA = 0.85) , and (b) a Bessel beam (1,1) with $\theta = \mathrm{asin}(0.5)$.

Figure S1 shows the radial behavior of functions g_0, g_2, h_0, h_2 for the case of a focused plane wave excitation (Eq. (S16)) and a pure Bessel beam (1, 1), with $f_0 \propto (1 + \cos(\theta))J_0$, $f_1 \propto \sin(\theta)J_1$, $f_2 \propto (1 - \cos(\theta))J_2$. The left panel of Figure S1 shows that the $\mathbf{e}_R$ component, $|g_0|$ (orange curves), is dominant for the terms of

$\mathbf{P}^{3\omega}$, which do not depend on ε. It is expected from other θ-dependent factors multiplying each polarization in Eq. (S2), namely $(1+\cos\theta)$ for $\mathbf{e}_R$ and $(1-\cos\theta)$ for $\mathbf{e}_L$, for the case of Bessel beams, and more complicated Eqs. (S17)-(S19) for the focused plane wave. Therefore, dominating contribution of ε-independent terms of TH response is $(3,1)$, and for ε^3-proportional terms, it is $(-3,-1)$. One can see that for a Bessel beam only h_0 (orange curves) would have maximum in the center, which coincides with the results for a plane wave, depicted in Figure S1a. The right panel in Figure S1 corresponds to the terms, proportional to ε, where the contribution of $(1,1)$ is dominant, and to ε^2, with dominance of $(-1,-1)$.

E Expression for $\varepsilon(\gamma)$

Electric field $\mathbf{E}'$, transmitted through a wave plate with a given retardance angle τ and the angle γ between the y-axis and the fast axis can be written in terms of Jones vectors as follows [13, 14]:

$$\begin{pmatrix} E'_x \\ E'_y \end{pmatrix} = R(-\gamma)M(\tau)R(\gamma)\begin{pmatrix} E_x \\ E_y \end{pmatrix} \tag{S25}$$

where

$$M(\tau) = \begin{bmatrix} \exp(\mathrm{i}\tau) & 0 \\ 0 & 1 \end{bmatrix} \tag{S26}$$

and

$$R(\gamma) = \begin{bmatrix} \cos\gamma & -\sin\gamma \\ \sin\gamma & \cos\gamma \end{bmatrix} \tag{S27}$$

From Eq. (S25) for y-polarized input $\mathbf{E} = (0,1)^\mathsf{T}$ we have:

$$\begin{aligned} \mathbf{E}' = (1-\exp(\mathrm{i}\tau))\sin\gamma\cos\gamma\hat{\mathbf{x}}+ \\ +(\sin^2\gamma\exp(\mathrm{i}\tau)+\cos^2\gamma)\hat{\mathbf{y}} \end{aligned} \tag{S28}$$

Taking into account (S12) and (S13), we can rewrite $\mathbf{E}'$ in the form $\mathbf{e}_R + \varepsilon\mathbf{e}_L$:

$$\begin{aligned} &\mathbf{E}' \propto \\ &[-\mathrm{i}(\sin^2\gamma\exp(\mathrm{i}\tau)+\cos\gamma^2)+(1-\exp(\mathrm{i}\tau))\sin\gamma\cos\gamma]\mathbf{e}_R+ \\ &+[\mathrm{i}(\sin^2\gamma\exp(\mathrm{i}\tau)+\cos^2\gamma)+(1-\exp(\mathrm{i}\tau))\sin\gamma\cos\gamma]\mathbf{e}_L \end{aligned}$$

Finally, dividing by the coefficient in front of $\mathbf{e}_R$ and simplifying for ε we get:

$$\varepsilon(\gamma) = -\frac{\mathrm{i}\sin 2\gamma\cos\tau+\cos 2\gamma}{\sin 2\gamma\sin\tau+1}e^{-2\mathrm{i}\gamma} \tag{S29}$$

From this expression we can find the ellipticiy angle α and the ellipse inclination angle β in terms of γ and τ:

$$\sin 2\alpha = \frac{S_3}{S_0} = \frac{1-|\varepsilon|^2}{1+|\varepsilon|^2} = \sin 2\gamma\sin\tau \tag{S30}$$

$$\tan 2\beta = \frac{S_2}{S_1} = \frac{\mathrm{Im}\varepsilon}{\mathrm{Re}\varepsilon} = \frac{-\sin 2\gamma\cos 2\gamma(1-\cos\tau)}{\cos\tau\sin^2 2\gamma+\cos^2 2\gamma} \tag{S31}$$

Equations (S30), (S31) can be obtained considering the Mueller matrix of a Babinet-Soleil compensator with a y-polarized input [14].

F Schematic of the optical setup

Figure S2 shows a schematic of the optical setup used to demonstrate the generation of TH patterns (Figs. 2 and 3 from the main text). The elements GP2 and QWP2 were used when conducting polarization-resolved measurements. The removable beam splitter (BS) was used only to illuminate the sample when focusing on the surface of the a-Si film.

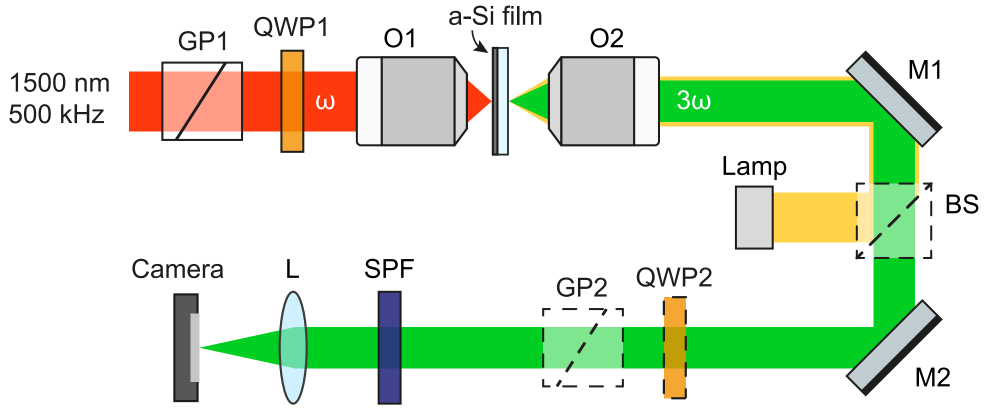

Figure S2: Schematic of the optical setup; here GP — Glan polarizer, M — mirror, L — lens, SPF — short pass filter, O — objective, BS — beam splitter.

G Polarization resolved measurements of TH signal

Figure S3 shows additional intensity patterns of the TH signal obtained in the polarization resolved experiment. In Fig. 3 of the main text are shown patterns obtained with pump ellipticity angles α of 43.9° and 37.9°.

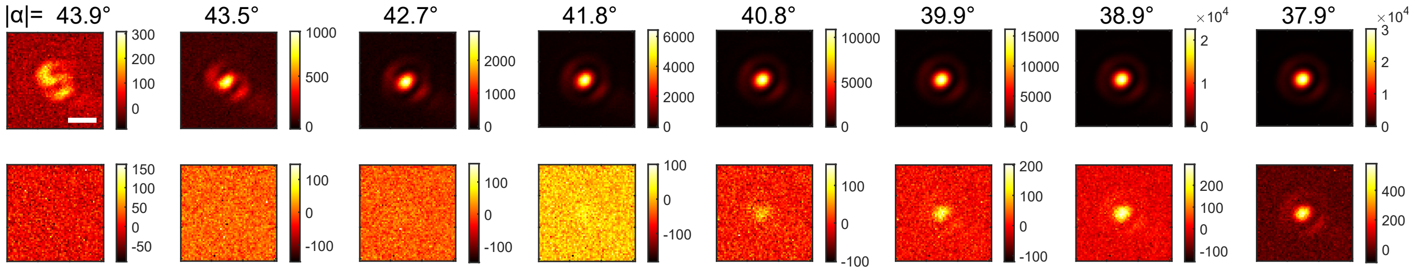

Figure S3: Polarization resolved measurements of TH signal, obtained when probing RCP (top panel) and LCP (bottom panel) light. Scale bar, 2 μm.

H Background TH generation by the optics of the setup

Figure S4 shows experimentally measured TH signals, when focusing on the silica substrate/air interface. One can see that in this case the signal intensity and its shape do not depend on the polarization of the pump. This behavior can be explained by the generation of parasitic TH in the optical elements of the setup. When focusing on the film, this signal is strongly attenuated, due to the high absorption of a-Si at the TH frequency (>40 dB μm^{-1} at 500 nm [15]),

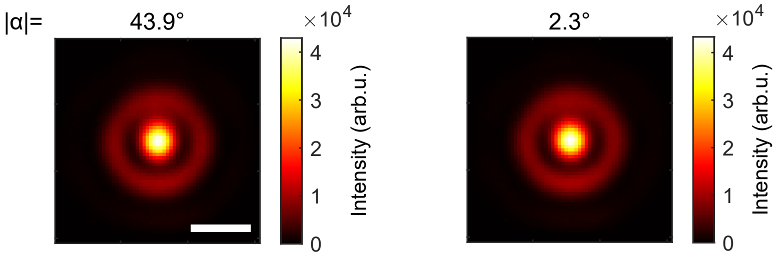

Figure S4: TH signal collected when focusing on substrate/air interface with ellipticity angle of the pump equal to 43.9° (close to circular) and 2.3° (close to linear). TH signal shape and intensity do not depend on the pump polarization. Scale bar, 2 μm.

I Off-axis interferometric measurements

Figure S5 shows the results of experimental measurements and numerical simulations of THG patterns generated by the thin film, as well as the patterns resulting from interference with a plane wave at the TH frequency. To provide a reference, we also add numerical simulations of patterns produced by a pure CP input pump. The appearance of two additional interference fringes can be observed, which manifests the generation of a beam with TAM = 3.

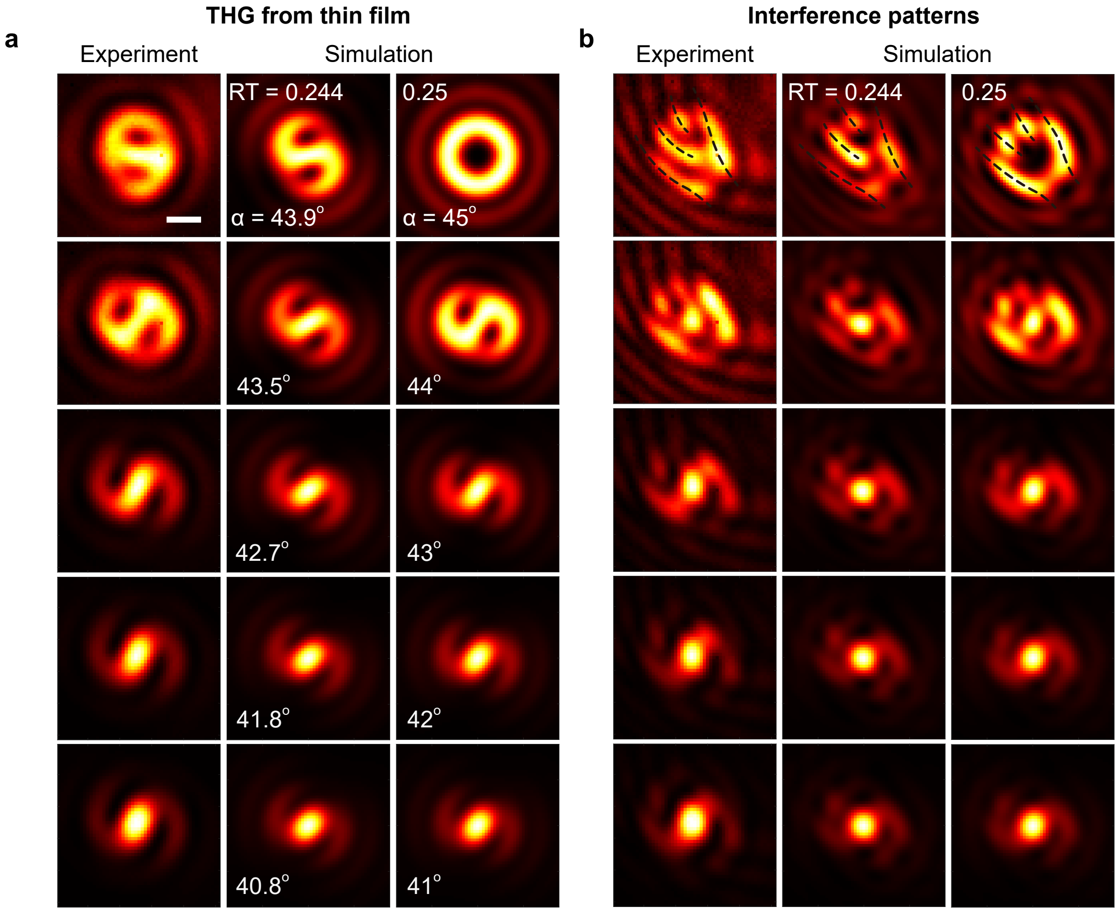

Figure S5: (a) Experimental measurements of TH patterns generated by a thin a-Si film, and numerically calculated patterns for input beams with imperfect (retardance RT=0.244) and ideal (RT = 0.25) QWPs. (b) Experimental measurements of interference patterns and numerically calculated patterns.

In the numerical simulation of the interference pattern, we consider the interference between the field generated by the thin film, $E^{\rm TH}$, and a y-polarized plane wave incident on the camera at an angle θ. We also account for the presence of a quadratic phase profile introduced during propagation through the collection optics. Therefore, the numerically calculated interference patterns are given by the following equation:

$$I_{\rm int} = \left| E_y^{\rm TH} - {\rm i} A \exp\left\{ \frac{2\pi {\rm i}}{\lambda}(x \sin\theta + z\cos\theta) \right\} \exp\left\{ \frac{2\pi {\rm i}}{F}[(x-x_0)^2 + (y-y_0)^2] \right\} \right|^2 + |E_x^{\rm TH}|^2 \tag{S32}$$

where $E_x^{\rm TH}$ and $E_y^{\rm TH}$ are the x and y components of the calculated electric field at TH, θ is the angle of incidence of the interfering wave, F is the quadratic phase parameter, and x_0, y_0 are the displacements of the quadratic profile origin. In the simulation shown above, the numerical values of the parameters were: $x_0 = 20$ μm, $y_0 = 115$ μm, $F = 1.32 \times 10^4$ μm^2, and $\theta = 0.7°$.

In the figure below a schematic of the setup we built for the interferometric measurements is illustrated. Here the TH signal generated by an a-Si film under tightly focused pump excitation, propagates in the "vortex" arm of the setup. To generate a reference TH signal we use a-Si grating metasurface (MS), featuring a narrow resonance near the excitation wavelength (1500 nm). Temporal overlap of the TH signals is controlled by a delay line, represented by the mirrors M6 and M7 mounted on a translational stage. Intensities of the generated signals were controlled independently by pump attenuators of corresponding arm, composed of half wave-plates (HWP1, HWP2) and Glan polarizers (GP1, GP2).

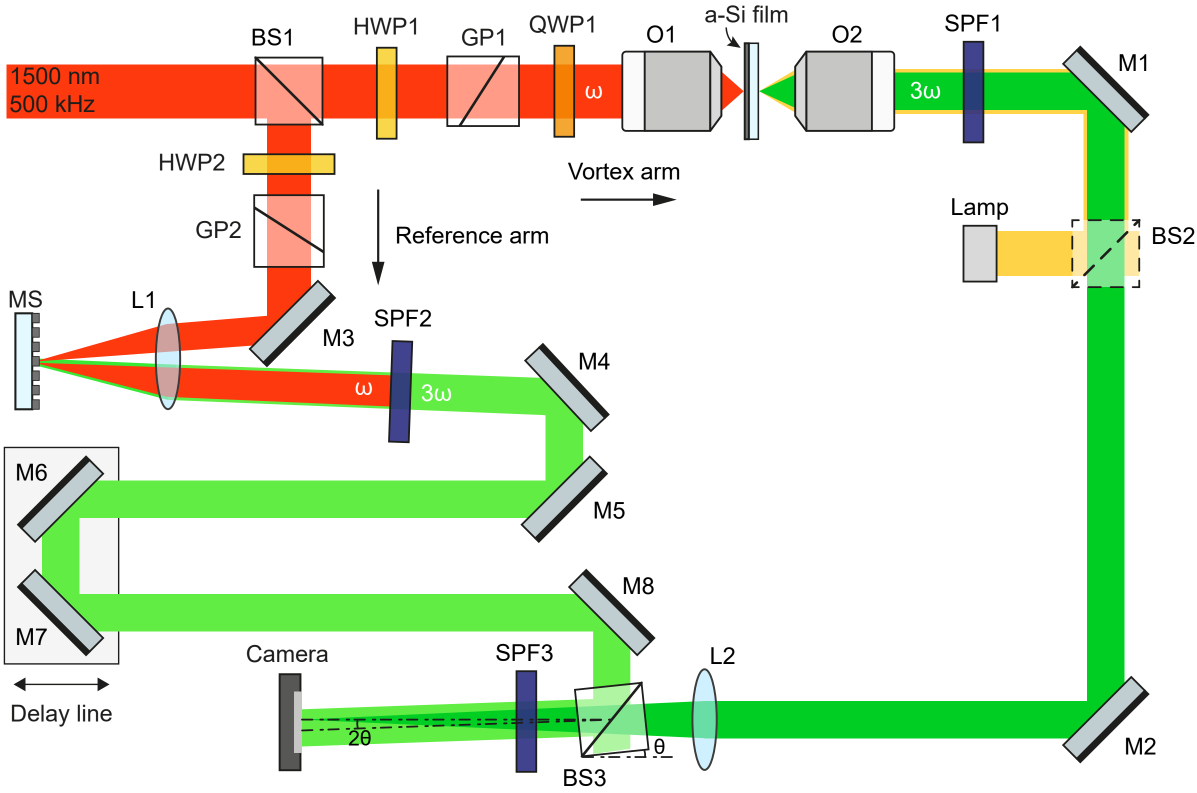

Figure S6: Schematic of the off-axis interferometry setup; here GP — Glan polarizer, M — mirror, L — lens, SPF — short pass filter, O — objective, BS — beam splitter, MS — metasurface.

We note that the conducted interferometric measurements were successfully performed after 11 months using the same sample, which indicates a slow degradation of the material properties.

J Quantitative analysis of the TAM composition

To quantitatively analyze the numerically calculated fields, we perform a fit of the numerically calculated TH field distributions using the set of terms presented in Eq. (4). Specifically, we determine the real valued weights a_i in the following

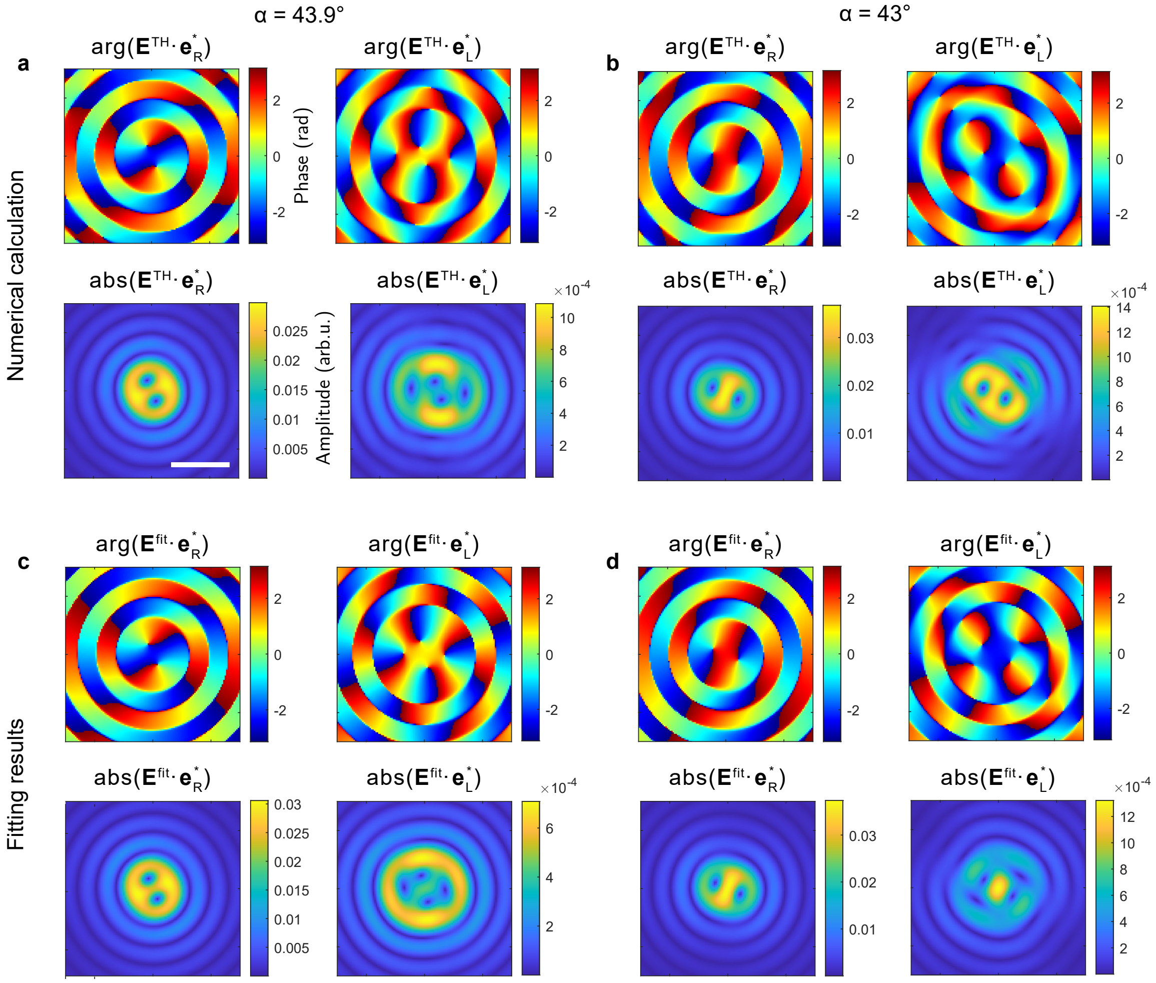

Figure S7: Numerical calculation of TH fields generated by a thin nonlinear layer and fitting results for excitation with input elipticity angle (a, c) $\alpha = 43.9°$ and (b, d) $\alpha = 43°$. Scale bar, 2 μm.

expression:

$$\mathbf{E}^{\text{fit}} = a_1(3,1) + a_2(1,1) + a_3(-3,-1) + a_4(-1,-1) \tag{S33}$$

where the terms (m, λ) are defined as follows (Eq. (3), main text):

$$(m, \lambda) = \int_0^{\theta_{\max}} \sin(\theta)\, c_{m\lambda}(\theta)\, \mathcal{B}^{k\theta}_{m\lambda}(\rho, \varphi, z)\, \mathrm{d}\theta \tag{S34}$$

Here, $c_{m\lambda}(\theta)$ is a complex weight coefficient, and $B^{k\theta}_{m\lambda}(\rho, \varphi, z)$ denotes a Bessel beam of well-defined helicity. In order to compute the numerical values of the $c_{m\lambda}$ coefficients, we calculate the projection of the corresponding Bessel beam on the in-plane components of the calculated TH field, or explicitly:

$$c_{m\lambda} = A\mathcal{B}^{k\theta *}_{m\lambda} \cdot \mathbf{E}^{\text{TH}} \tag{S35}$$

Additionally, for each (m, λ) term we optimize the angles of frame rotation β_i, i.e., $\varphi \to \varphi + \beta_i$, to account for the phase difference between the RCP and LCP components, arising in polarization components $P_i^{3\omega}$, due to the propagation of the pump beam (see Eqs. (S22), (S24)).

To calculate the expansion weights a_i, we minimize the target function defined as follows [16]:

$$FT = \left\| \left(\mathbf{E}^{\text{fit}} - \mathbf{E}^{\text{TH}}\right) \cdot \mathbf{e}_R^* \right\|^2 + \left\| \left(\mathbf{E}^{\text{fit}} - \mathbf{E}^{\text{TH}}\right) \cdot \mathbf{e}_L^* \right\|^2 \tag{S36}$$

Figure S7 shows the comparison between the numerically calculated TH electric field projections, and the results obtained by fitting using Bessel beams of well-defined helicity.

The fitting error for the right- and left-circularly polarized (RCP and LCP) components is defined as:

$$\sigma_{R,L} = \frac{\left\|(\mathbf{E}^{\text{fit}} - \mathbf{E}^{\text{TH}}) \cdot \mathbf{e}^*_{R,L}\right\|^2}{\left\|\mathbf{E}^{\text{TH}} \cdot \mathbf{e}^*_{R,L}\right\|^2}, \tag{S37}$$

which gives values of

$$\sigma_R = 0.0012 \quad \text{and} \quad \sigma_L = 0.2033 \quad \text{with} \quad \alpha = 43.9^\circ, \tag{S38}$$

and

$$\sigma_R = 0.0048 \quad \text{and} \quad \sigma_L = 0.3050 \quad \text{with} \quad \alpha = 43^\circ. \tag{S39}$$

The fitting parameters for the corresponding terms are given in the table:

		1	2	3	4
$\alpha = 43.9^\circ$	a_i	0.89984±0.23e-3	0.32723±0.22e-3	0.00044±0.26e-3	0.00616±0.22e-3
	β_i (rad)	-0.12	-1.08	-0.95	-2.05
$\alpha = 43^\circ$	a_i	0.80896±0.26e-3	0.52144±0.25e-3	0.00072±0.29e-3	0.01844±0.25e-3
	β_i (rad)	-0.12	-1.04	-0.99	-2.05

Considering the absolute values of the weights for the two ellipticity angles, normalized by their respective maximum values, we obtain the ratios $1 : 0.36 : 0.49 \times 10^{-5} : 0.68 \times 10^{-2}$ for $\alpha = 43.9^\circ$, and $1 : 0.64 : 0.89 \times 10^{-5} : 0.023$ for $\alpha = 43^\circ$. Comparing the weights, one can observe that decreasing the ellipticity angle leads to an increase in the amplitudes of the components with different total angular momentum.

We should note that the projections of oppositely polarized TH electric fields in the fit show relatively weak correspondence with the calculated distributions. This discrepancy can be attributed to the breaking of duality transformations in the nonlinear processes, as described in the main text. The change in the helicity of the generated TH signal can be accounted for in the fit by introducing additional terms with opposite helicity, resulting in the following equality

$$\begin{aligned}\mathbf{E}^{\text{fit}} &= a_1(3,1) + a_2(1,1) + a_3(-3,-1) + a_4(-1,-1) + \\ &+ a_5(3,-1) + a_6(1,-1) + a_7(-3,1) + a_8(-1,1),\end{aligned} \tag{S40}$$

which provides a more accurate correspondence for the LCP projection, namely,

$$\sigma_R = 0.0011 \quad \text{and} \quad \sigma_L = 0.0063 \quad \text{with} \quad \alpha = 43.9^\circ, \tag{S41}$$

and

$$\sigma_R = 0.0047 \quad \text{and} \quad \sigma_L = 0.0160 \quad \text{with} \quad \alpha = 43^\circ. \tag{S42}$$

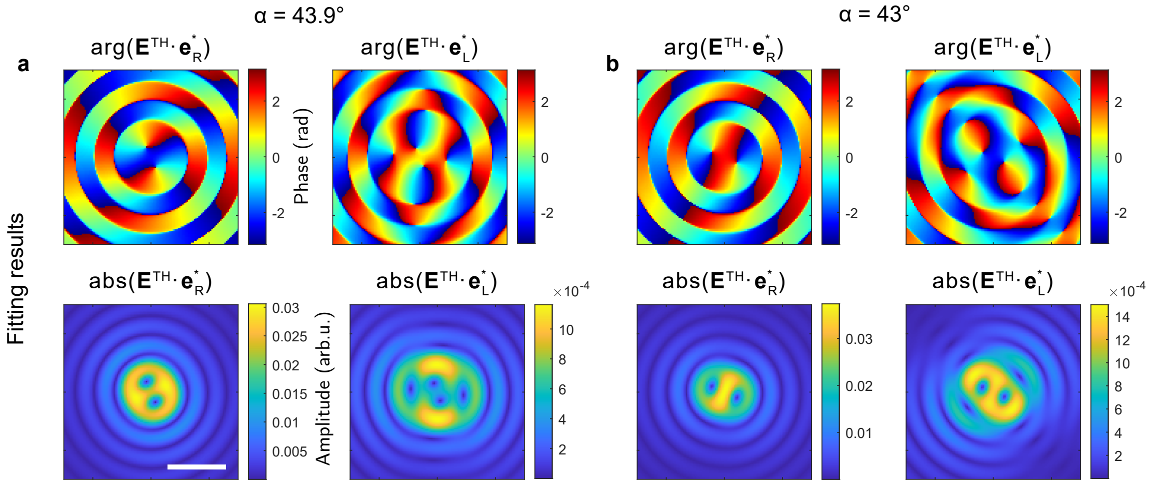

Figure S8: Fitting results for excitation with input elipticity angle (a) $\alpha = 43.9^\circ$ and (b) $\alpha = 43^\circ$ using extended model. Scale bar, 2 μm.

The field distributions for the extended fit model are shown in Figure S8, and the fitting parameters are provided in the table below:

$\alpha = 43.9°$	a_i	1	2	3	4
		0.89944±0.19e-3	0.32650±0.19e-3	0.00025±0.22e-3	0.00640±0.19e-3
		5	6	7	8
		0.00687±0.20e-3	0.01750±0.19e-3	0.00227±0.22e-3	0.01159±0.22e-3
$\alpha = 43°$	a_i	1	2	3	4
		0.80860±0.19e-3	0.52020±0.19e-3	0.00042±0.21e-3	0.01815±0.18e-3
		5	6	7	8
		0.00611±0.20e-3	0.02794±0.19e-3	0.00363±0.22e-3	0.01071±0.21e-3

K TH generation under excitation through low-NA objective

Figure S9a presents numerical simulations and experimental measurements of TH signal distributions acquired using an objective with a numerical aperture of 0.13 (Olympus LMPlanFLN 5x). In contrast to the results obtained with the high-NA objective (NA = 0.85) discussed in the main text, neither the formation of two-lobe profiles nor the emergence of central minima is observed. This behaviour can be attributed to the pronounced sensitivity of the TH beam structure to the polarization purity of the excitation beam under weak focusing conditions. This is evidenced by different amplitudes of RCP and LCP components of polarization $\mathbf{P}^{3\omega}$, shown in Figure S9b. One can note that the amplitude of h_0 component, featuring no phase singularity in the dominant polarization (see Eq. (S24)), increases by 2 orders of magnitude, compared to focusing through a NA=0.85 objective (see Figure S1a). Therefore, the observation of the vortex component requires suppression of this term by proportional increasing the purity of input beam (i.e., lowering values of ε).

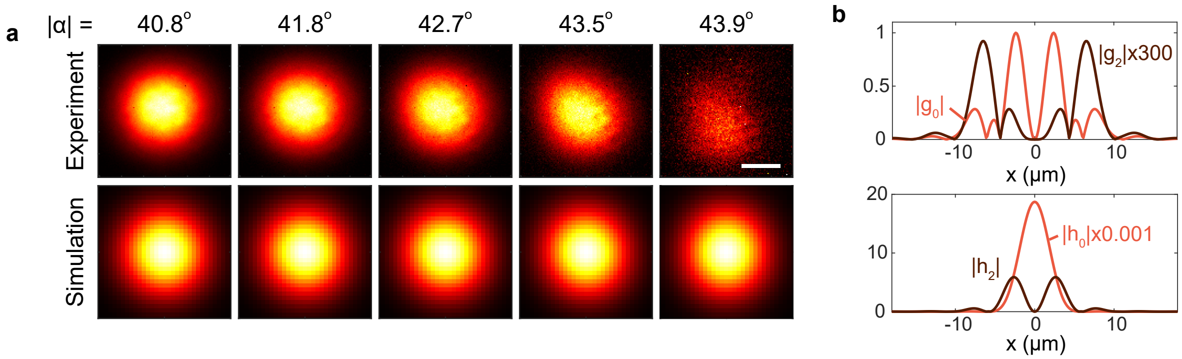

Figure S9: (a) Experimental measurements and numerical simulations of TH intensity distributions generated by a thin a-Si layer under excitation with a low-NA objective. (b) Calculated components of polarization $\mathbf{P}^{3\omega}(z_0 = 0)$ for pump beam focusing using a lens with NA = 0.15. Scale bar, 2 μm.

L Numerical simulation of TH generation with and without the substrate

Figure S10 shows a numerical simulation of TH generation by a focused laser beam when either taking into account the presence of the air/silica substrate interface or neglecting it. Comparing the upper and bottom panels, one can see that the presence of the interface does not significantly alter the TH patterns. Therefore, for simplicity, in the main text we discuss the case without the air/substrate interface, shown in Figure S10b.

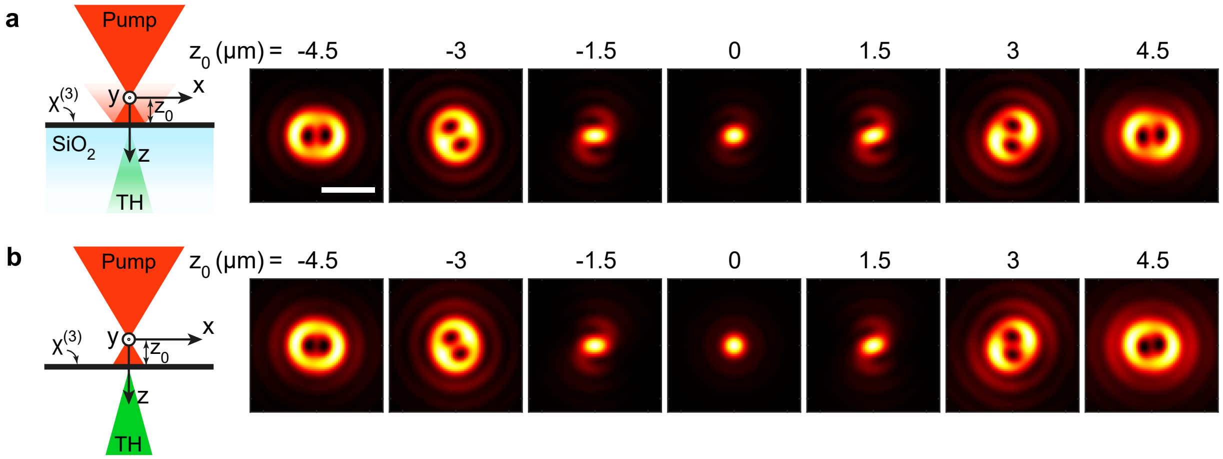

Figure S10: a) TH intensity patterns calculated numerically, taking into account the presence of the air/substrate interface, b) patterns calculated for the nonlinear layer placed in the air; z_0 is the defocusing parameter. Scale bar, 2 μm.

M THG conversion efficiency

We have calculated the THG conversion efficiency under plane wave illumination at variable angle of incidence. The pump is tuned at 1.5 μm, and we consider three different polarization states of the pump: s-polarization, p-polarization, left-handed circular polarization (LHCP). The silicon film has thickness of 1 μm and refractive index dispersion taken from [17]. The third-order susceptibility used in the simulation is $\chi^{(3)} = 2.45 \cdot 10^{-19}$ m^2/V^2 [18], whereas a pump peak intensity of 1 GW/cm^2 is assumed. While for s- and p-polarized pumps the THG conversion efficiency shows its maximum peak $\frac{P_{3\omega}}{P_\omega} \approx 10^{-7}$ when the pump is at normal incidence, for circularly polarized pump the THG is forbidden by the tensor symmetry at normal incidence. However, it rapidly increases, reaching a maximum efficiency of $5 \cdot 10^{-10}$ at an angle of incidence approximately equal to 58°. Since the numerical aperture of the lens used to focus the pump is 0.85 (corresponding to a $\theta_{\max} \approx 58°$), the maximum THG that we may expect in our experimental setup for pure circular polarization is $5 \cdot 10^{-10}$. When the pump is elliptically polarized, the maximum efficiency may increase by more than 2 orders of magnitude.

References

[1] Wu-Ki Tung. *Group Theory in Physics*. World Scientific Publishing Company, Singapore, 1985. doi:10.1142/0097.

[2] A. I. Akhiezer and V. B. Berestetsky. *Quantum Electrodynamics*. Interscience Publishers, Hoboken, NJ, USA, 1965.

[3] Peter Woit. *Quantum Theory, Groups and Representations: An Introduction*. Springer, Berlin, Germany, 2017. URL: https://www.math.columbia.edu/~woit/QM/qmbook.pdf.

[4] Ivan Fernandez-Corbaton. *Helicity and duality symmetry in light matter interactions: Theory and applications*. PhD thesis, Macquarie University, 2014. arXiv: 1407.4432. doi:10.48550/arXiv.1407.4432.

[5] S. Hacyan and R. Jáuregui. A relativistic study of Bessel beams. *J. Phys. B: At. Mol. Opt. Phys.*, 39(7):1669, 2006. doi:10.1088/0953-4075/39/7/009.

[6] Andrei Afanasev, Carl E. Carlson, and Asmita Mukherjee. Off-axis excitation of hydrogenlike atoms by twisted photons. *Phys. Rev. A*, 88(3):033841, 2013. doi:10.1103/PhysRevA.88.033841.

[7] Ivan Fernandez-Corbaton, Xavier Zambrana-Puyalto, and Gabriel Molina-Terriza. Helicity and angular momentum: A symmetry-based framework for the study of light-matter interactions. *Phys. Rev. A*, 86(4):042103, 2012. doi:10.1103/PhysRevA.86.042103.

[8] Robert Boyd. *Nonlinear Optics*. Academic Press, Cambridge, MA, USA, Dec 2002. URL: https://www.elsevier.com/books/nonlinear-optics/boyd/978-0-12-121682-5.

[9] Anastasia Nikitina and Kristina Frizyuk. Achiral Nanostructures: Perturbative Harmonic Generation and Dichroism Under Vortex and Vector Beams Illumination. *Adv. Opt. Mater.*, 12(25):2400732, 2024. doi:10.1002/adom.202400732.

[10] Kristina Frizyuk, Elizaveta Melik-Gaykazyan, Jae-Hyuck Choi, Mihail I. Petrov, Hong-Gyu Park, and Yuri Kivshar. Nonlinear Circular Dichroism in Mie-Resonant Nanoparticle Dimers. *Nano Lett.*, 21(10):4381–4387, 2021. doi:10.1021/acs.nanolett.1c01025.

[11] Bernard Richards and Emil Wolf. Electromagnetic diffraction in optical systems, ii. structure of the image field in an aplanatic system. *Proceedings of the Royal Society of London. Series A. Mathematical and Physical Sciences*, 253(1274):358–379, 1959. doi:10.1098/rspa.1959.0200.

[12] Rishi Kant. An Analytical Solution of Vector Diffraction for Focusing Optical Systems. *J. Mod. Opt.*, 1993. URL: https://www.tandfonline.com/doi/abs/10.1080/09500349314550341.

[13] William A. Shurcliff. *Polarized Light*. Harvard University Press, Cambridge, MA, USA, 2013. doi:10.4159/harvard.9780674424135.

[14] Edward Collett. Polarized light. fundamentals and applications. *Optical Engineering*, 1992.

[15] Yoshihiro Hishikawa, Noboru Nakamura, Shinya Tsuda, Shoichi Nakano, Yasuo Kishi Yasuo Kishi, and Yukinori Kuwano Yukinori Kuwano. Interference-Free Determination of the Optical Absorption Coefficient and the Optical Gap of Amorphous Silicon Thin Films. *Jpn. J. Appl. Phys.*, 30(5R):1008, 1991. doi:10.1143/JJAP.30.1008.

[16] P. Bevington and D.K. Robinson. *Data Reduction and Error Analysis for the Physical Sciences*. McGraw-Hill Education, 2003. URL: https://books.google.it/books?id=0poQAQAAIAAJ.

[17] Daniel Franta, David Nečas, Lenka Zajíčková, Ivan Ohlídal, Jiří Stuchlík, and Dagmar Chvostová. Application of sum rule to the dispersion model of hydrogenated amorphous silicon. *Thin Solid Films*, 539:233–244, 2013. doi:10.1016/j.tsf.2013.04.012.

[18] Kirill Koshelev, Yutao Tang, Kingfai Li, Duk-Yong Choi, Guixin Li, and Yuri Kivshar. Nonlinear Metasurfaces Governed by Bound States in the Continuum. *ACS Photonics*, 6(7):1639–1644, 2019. doi:10.1021/acsphotonics.9b00700.
